# Alterations of the gut microbiota in patients with schizophrenia

**DOI:** 10.3389/fpsyt.2024.1366311

**Published:** 2024-03-26

**Authors:** Zhuocan Li, Xiangkun Tao, Dongfang Wang, Juncai Pu, Yiyun Liu, Siwen Gui, Xiaogang Zhong, Dan Yang, Haipeng Zhou, Wei Tao, Weiyi Chen, Xiaopeng Chen, Yue Chen, Xiang Chen, Peng Xie

**Affiliations:** ^1^ National Health Commission (NHC) Key Laboratory of Diagnosis and Treatment on Brain Functional Diseases, The First Affiliated Hospital of Chongqing Medical University, Chongqing, China; ^2^ Jinfeng Laboratory, Chongqing, China; ^3^ Chongqing Institute for Brain and Intelligence, Chongqing, China; ^4^ Department of Neurology, The First Affiliated Hospital of Chongqing Medical University, Chongqing, China; ^5^ College of Basic Medicine, Chongqing Medical University, Chongqing, China

**Keywords:** schizophrenia, gut microbiota, microbial biomarkers, microbial diversity, gut-brain-axis

## Abstract

**Introduction:**

Schizophrenia is a complex psychiatric disorder, of which molecular pathogenesis remains largely unknown. Accumulating evidence suggest that gut microbiota may affect brain function via the complex gut-brain axis, which may be a potential contributor to schizophrenia. However, the alteration of gut microbiota showed high heterogeneity across different studies. Therefore, this study aims to identify the consistently altered gut microbial taxa associated with schizophrenia.

**Methods:**

We conducted a systematic search and synthesis of the up-to-date human gut microbiome studies on schizophrenia, and performed vote counting analyses to identify consistently changed microbiota. Further, we investigated the effects of potential confounders on the alteration of gut microbiota.

**Results:**

We obtained 30 available clinical studies, and found that there was no strong evidence to support significant differences in α-diversity and β-diversity between schizophrenic patients and healthy controls. Among 428 differential gut microbial taxa collected from original studies, we found that 8 gut microbial taxa were consistently up-regulated in schizophrenic patients, including Proteobacteria, Gammaproteobacteria, *Lactobacillaceae*, *Enterobacteriaceae*, *Lactobacillus*, *Succinivibrio*, *Prevotella* and *Acidaminococcus*. While 5 taxa were consistently down-regulated in schizophrenia, including *Fusicatenibacter*, *Faecalibacterium*, *Roseburia*, *Coprococcus* and *Anaerostipes*.

**Discussion:**

These findings suggested that gut microbial changes in patients with schizophrenia were characterized by the depletion of anti-inflammatory butyrate-producing genera, and the enrichment of certain opportunistic bacteria genera and probiotics. This study contributes to further understanding the role of gut microbiota in schizophrenia, and developing microbiota-based diagnosis and therapy for schizophrenia.

## 1 Introduction

Schizophrenia is a highly heterogeneous and devastating chronic psychiatric disorder, characterized with abnormal mental functions and disturbed behaviors (1–3). The global prevalence of schizophrenia is estimated to range from 0.5% to 1% (4), significantly impacting both the social functioning and life expectancy of individuals (5, 6). Currently, the underlying pathogeneses of schizophrenia have yet to be completely elucidated (7). Clinically, there are no robust biomarkers to aid in diagnosis or prognosis, and the diagnosis of schizophrenia primarily relies on the subjective identification of clinical symptoms of patients (8). Thus, it is of significant clinical value to identify molecular biomarkers sensitive to the pathological processes of schizophrenia. Additionally, the current antipsychotics have severe limitations, including limited amelioration in symptoms and severe neurological and metabolic side effects (9). As a consequent, there is a pressing need for novel therapies to treat schizophrenia.

The human gut is inhabited by a complex and metabolically active microbial ecosystem (10, 11). In recent years, a number of studies have demonstrated the significant role of gut microbiota in the pathogenesis of schizophrenia via the intricate “microbiota-gut-brain” (MGB) axis (12, 13). The MGB establishes a bidirectional communication system between the central nervous system and gut microbiota, mediated by neural, immune, and endocrine pathways, thereby exerting influences on brain function (14, 15). Emerging evidence have demonstrated changes in the composition of gut microbiota between healthy individuals and patients with mental disorders (16). For instance, some studies have reported alterations in bacterial taxa among patients with schizophrenia, including a reduction in the relative abundance of short-chain fatty acid (SCFA) producing bacteria and an increase in pathogenic bacteria (17). Moreover, gut microbiota can synthesize metabolites (such as 5-hydroxytryptamine, kynurenine, and indole derivatives) that may impact on the central nervous system, which have been demonstrated to be involved in the pathogenesis of schizophrenia (18–20). In addition, preclinical studies have indicated that fecal microbiota transplants from patients with schizophrenia resulted in the development of schizophrenic-like behaviors in germ-free mice (21–23). These evidences suggest that schizophrenia may be associated with a distinct pattern of microbial perturbations. Therefore, the identification of key microbial taxa may contribute to both understanding etiology and identifying clinically useful biomarkers, as well as new targeted treatment strategies (24).

With the rapid development of high-throughput technologies, such as metagenomic sequencing, increasing studies have investigated the changes of the gut microbiota in patients with schizophrenia. However, results from these studies showed high heterogeneity due to the differences in the patient population, e.g. the sample size (25), disease severity (26), age (27), use of drugs (28), residential diet (29), and diagnostic criteria (30, 31). Although there are several systematic reviews summarizing gut microbial alterations in schizophrenia, the lack of quantitative analysis hinders the identification of microbial markers across different studies (32).

Therefore, the aim of this study is to assess the perturbations in gut microbial diversity and taxonomy in patients with schizophrenia compared to controls, and to identify consistently altered gut microbiota in schizophrenia by synthesizing the totality of evidence from gut microbiome studies.

## 2 Materials and methods

### 2.1 Search strategy

This study was conducted by following the Preferred Reporting Items for Systematic reviews and Meta-analyzes (PRISMA) statement recommendations (33). We conducted a comprehensive search in PubMed, Cochrane Library, Embase and Web of Science databases up until July 1st, 2023 to identify studies reporting alterations in the gut microbiome among patients with schizophrenia.

In accordance with the PICO criteria, population were patients diagnosed with schizophrenia; intervention was drug or untreated; comparison was control or placebo; outcomes were symptom, gut microbiota, feces, 16S rRNA or metagenome. The keywords used to develop the search strategy were shown in **Supplementary Table S1**, and the details of the search strategy were listed in **Supplementary Table S2**.

### 2.2 Data inclusion and exclusion

The eligibility of all studies was independently screened and evaluated by two researchers (Li Z and Tao X), with intervention from a third reviewer (Wang D) in case of any disagreements.

The studies eligible for data extraction were required to satisfy the following criteria: 1) original human research; 2) utilization of standardized diagnostic criteria for schizophrenia, such as The Diagnostic and Statistical Manual of Mental Disorders, fourth/fifth edition (DSM-IV/V) or International Classification of Diseases (ICD-10); 3) implementation of high-throughput technologies, e.g. 16S rRNA amplicon sequencing and metagenomic sequencing; 4) reporting statistically significant disparities in diversity indices (α and β diversity) or gut microbiota abundance (increased or decreased) between the disease group and healthy control group.

The exclusion criteria included animal studies, secondary analyzes (such as meta-analysis), literature reviews, and conference abstracts.

### 2.3 Assessment of study quality

The Newcastle-Ottawa quality assessment scale (NOS) was used to assess study quality by two researchers (Li Z and Tao X). The NOS assigns a maximum of 9 points based on three quality parameters, including selection, comparability, and outcome. According to the NOS grading in previous reviews, we classified studies as high (<5 stars), moderate (5–7 stars) or low risk of bias (≥8 stars) (34).

### 2.4 Data extraction

Data extraction was performed by Li Z using a pre-determined form, which underwent independent validation by another two researchers (Tao X and Wang D). The form documents fundamental information of the study (study name, title, diagnostic criteria for schizophrenia, organism, intervention, sample type, sample size, age, sex, BMI, sequencing method and platform, amplicon region, original data availability), and the information of the differential microbiota reported by the studies (microbiota name, classification level, Up/Down regulated, NCBI taxonomy ID, lineage, comparison groups, diversity assessment/alteration).

The present study exclusively focused on the identification of differential gut microbiota between the disease group and the healthy control group based on statistical significance criteria stipulated by each individual study. The gut microbiota extracted were across six classification levels, including phylum, class, order, family, genus, and species.

### 2.5 Subgroups comparisons

To investigate potential factors influencing gut microbiota, we further stratified the differential microbiota data based on the total score of Positive and Negative Syndrome Scale (PANSS), sequencing method, intervention type, country of residence, and age. Subsequent statistical analyzes were performed within each subgroup category and compared between subgroups within the same category or between a subgroup and the entire population.

### 2.6 Statistical analysis

Theoretically, the optimal approaches for integrating differential microbiome data were combining the mean values, *p*-values, or raw data from each study. However, conducting a meta-analysis proved to be challenging due to a lack of mean values, *p*-values, or raw data in most original studies. Consequently, we performed a vote-counting method to analyze whether microbiota were consistently up- or down-regulated across studies. The vote counting method can facilitate the identification of candidate biomarkers that are likely to be validated by independent testings (35).

Considering that the reporting frequency of each microbiota can be potentially diluted, we performed statistical analysis on the differential microbiota that were reported three or more times, designating them as “candidate microbiota”. During this process, each microbial taxon was assigned a weight of “+1” or “-1” when it was reported significantly up-regulated or down-regulated, respectively. The vote counting statistic (VCS) for each candidate taxon was calculated by summing the individual scores. Higher or lower VCS values indicated more studies reporting significant up-regulation or down-regulation of the candidate microbiota, respectively.

We used binomial distributions to assess whether the up-regulation or down-regulation of each candidate microbiota was statistically significant, assuming a probability of 50% for each taxon to be up- or down-regulated in each study (36). The binomial tests were conducted using the binom.test function in R (v 4.0.4, https://www.rproject.org/). One-sided *p*-values were calculated for candidate microbiota that were reported in more than three data sets. *P*-value less than 0.05 was considered to be statistically significant.

## 3 Results

### 3.1 Characteristics of included studies

**Figure 1:**
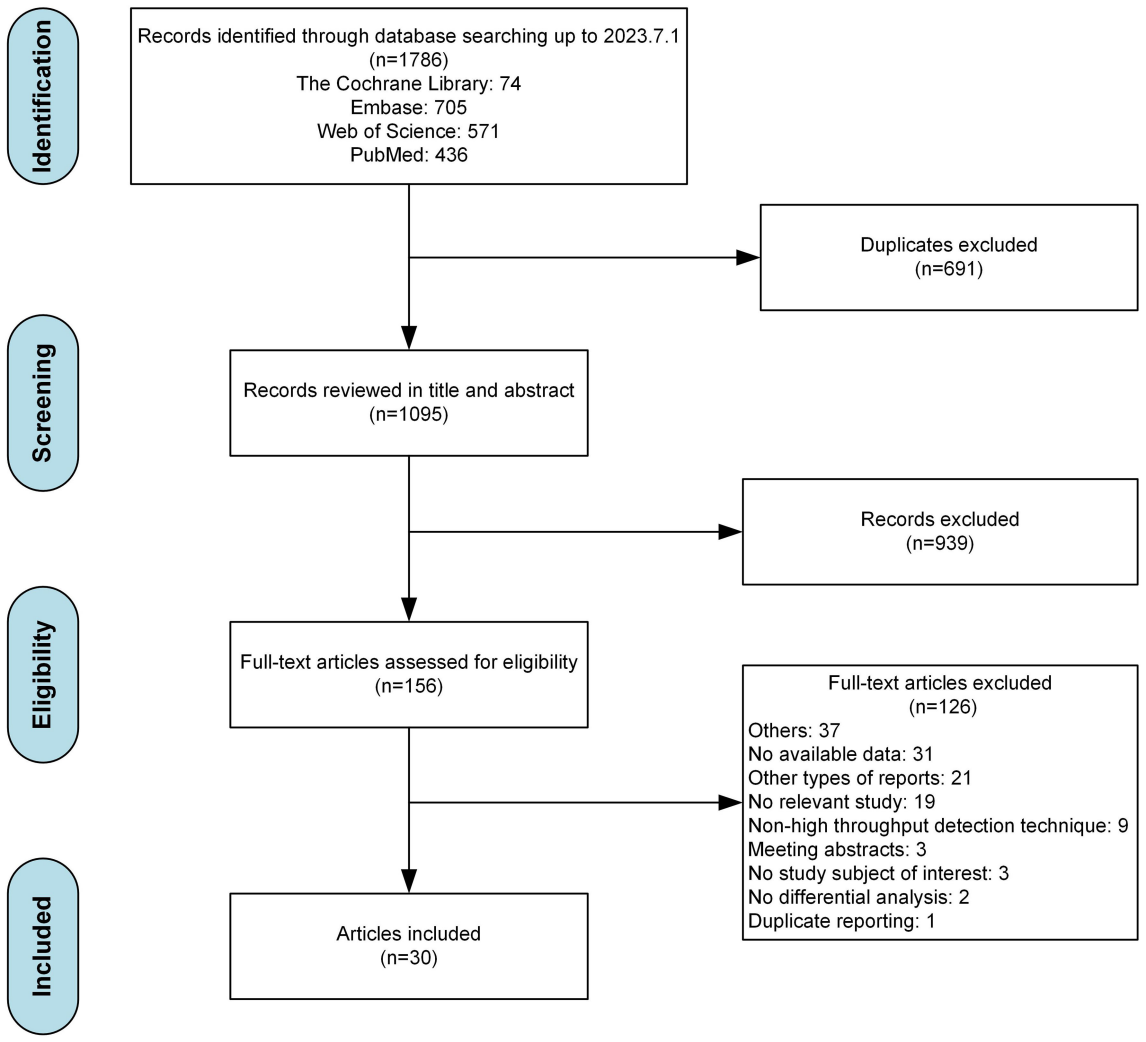
PRISMA flowchart.

The flowchart for data screening were illustrated as **Figure 1**. Among the 1786 records yielded by our database search, 1095 remained after the removal of duplicates. Based on our eligibility criteria, 156 articles were selected. Of these, 126 articles were excluded after a full-text screening (the detailed exclusion records were provided in **Supplementary Table S3**), resulting in 30 articles included. After conducting a quality evaluation using NOS as indicated in **Supplementary Table S4**, it was determined that 16 out of the 30 studies (53.3%) were classified as having a moderate risk of bias, while the remaining 14 studies (46.7%) were classified as having a low risk of bias.

Of the 30 eligible studies, there were 22 conducted in China, 2 in United States, and the remaining 6 studies in other countries including India, Australia, Denmark, Poland, Germany, and Italy. A total of 2001 patients with schizophrenia (1043 males and 868 females) and 1694 healthy controls (790 males and 833 females) were obtained, with one study [Ling Z 2022 (37)] did not report the sex ratio of the subjects. The sample size of each study ranged from 3 to 132. There were six studies involving drug treatment, including risperidone (3/6, 50%), clozapine (1/6, 16.7%), and unspecified antipsychotics (2/6, 33.3%). The diagnostic criteria for schizophrenia primarily based on DSM-IV (20/30, 66.7%) and DSM-V (5/30, 16.7%). Fifteen studies utilized the PANSS scale to assess the symptoms of patients with schizophrenia. In terms of sequencing methods, 16S rRNA amplicon sequencing was most widely used (22/30, 73.3%), followed by metagenomic sequencing (6/30, 20.0%), and 2 studies (6.7%) used both above methods. The detail information of the studies are provided in **Supplementary Table S5**.

### 3.2 Alterations of the microbial diversities in schizophrenia

**Figure 2:**
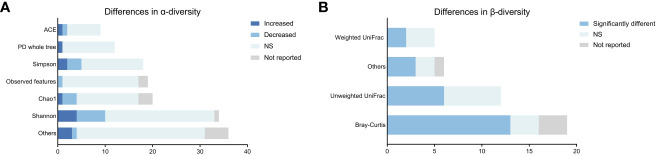
Differences in microbial α-and β-diversity in patients with schizophrenia compared to controls across studies. **(A)** Differences in α-diversity. **(B)** Differences in β-diversity.

For α-diversity analysis, there were 16 types of metrics used across 30 studies (**Supplementary Table S6**). As illustrated in **Figure 2A**, the most frequently reported metrics were ACE, PD whole tree, Simpson, Observed Features, Chao1 and Shannon. The majority of α-diversity analyzes (99/125, 79.2%) showed no statistically significant changes in patients with schizophrenia, while only 8.8% and 12.0% of analyzes demonstrated an increase or decrease of α-diversity, respectively. Overall, our synthesized data did not indicate strong evidence for the difference in the α-diversity of gut microbiota between schizophrenic patients and healthy controls.

For β-diversity analysis, a total of 8 algorithms were used across 30 studies (**Supplementary Table S6**). As shown in **Figure 2B**, the most frequently used algorithms were Bray-Curtis, unweighted UniFrac, and weighted UniFrac similarity. Dimension reduction approaches, such as principal component analysis (PCA), principal coordinate analysis (PCoA), and non-metric multidimensional scaling, were employed for β-diversity visualization. In our study, only 63.2% of the β-diversity analyzes (24/38) showed significant differences in the composition of gut microbiota in schizophrenia patients compared to controls. These findings did not strongly support that the gut microbiota composition of individuals with schizophrenia obviously differed from that of healthy controls.

### 3.3 Alterations of gut microbiota in schizophrenia

**Figure 3:**
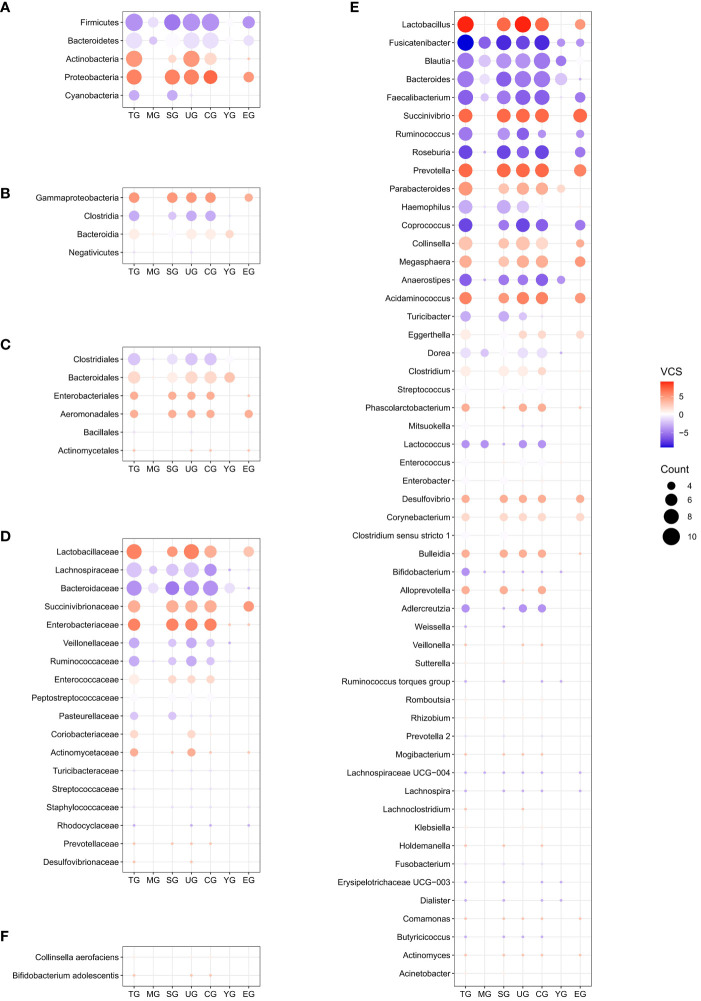
Distribution of candidate microbiota in the entire schizophrenic patient group and subgroups at phylum **(A)**, class **(B)**, order **(C)**, family **(D)**, genus **(E)**, and species **(F)** levels. The size of the circle represents the counting value, while the color of the circles corresponds to the magnitude of the vote counting statistic (VCS) value. The subgroup details and corresponding data are presented below. TG, entire patients group; MG, moderately ill group; SG, 16S rRNA amplicon sequencing group; UG, untreated group; CG, Chinese group; YG, youth group; EG, middle and elderly group; VCS, vote counting statistic.

After removing duplicates, a total of 428 differential gut microbial taxa from 30 studies were included in final analyzes (**Supplementary Table S7**). Eighty-eight candidate microbiota reported in three or more data sets were obtained, including 5 taxa at the phylum level, 4 at the class level, 6 at the order level, 18 at the family level, 53 at the genus level, and 2 at the species level (**Figure 3**; **Supplementary Table S8**). **Supplementary Figure S1** presents the lineage of these 88 candidate taxa. The differential genera were mainly assigned to *Lachnospiraceae* at family level, and mainly derived from Firmicutes, Actinobacteria and Proteobacteria at phylum level.

**Figure 4:**
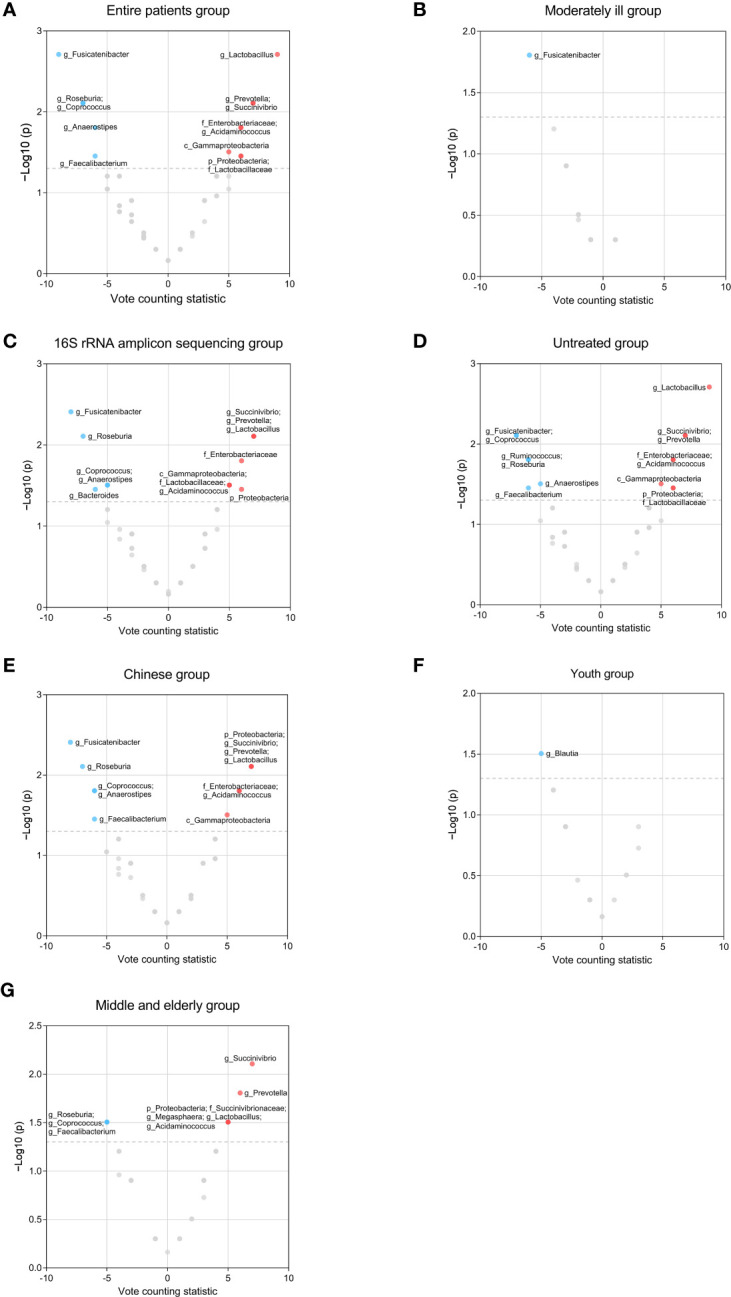
Volcano plots of candidate microbiota resulting from vote counting analyzes for the entire schizophrenic patient group and subgroups. The nodes represent candidate microbiota, while the x-axis illustrates the vote counting statistic and the y-axis displays the -log10 (*p*-value). The red dots indicate statistically significant up-regulation, while the blue dots represent statistically significant down-regulation. *p*< 0.05 were considered to be statistically significant. **(A)** Entire patient group. **(B)** Moderately ill group. **(C)** 16S rRNA amplicon sequencing group. **(D)** Untreated group. **(E)** Chinese group. **(F)** Youth group. **(G)** Middle and elderly group.

**Figure 5:**
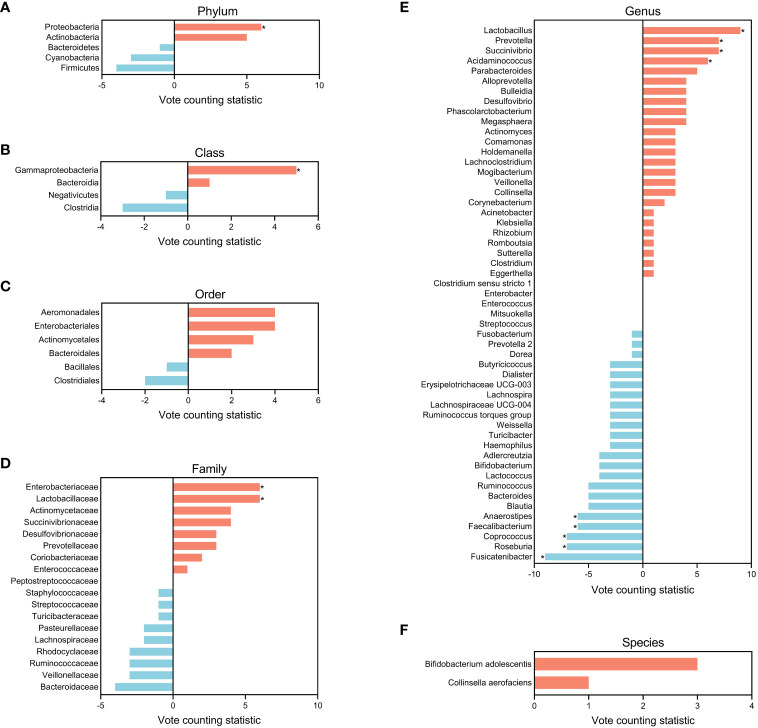
Bar plots of candidate microbiota for the entire schizophrenic patient group at phylum **(A)**, class **(B)**, order **(C)**, family **(D)**, genus **(E)**, and species **(F)** levels. The vote counting statistic for each candidate microbiota is represented by orange and blue bars. An asterisk (*) indicates *p* < 0.05.

To further identify gut microbial biomarkers for schizophrenia with consistent alterations compared to healthy controls, we conducted the vote counting analysis on the candidate taxa (for original statistics, see **Supplementary Table S8**). Ultimately, we identified a total of 13 taxa that showed consistent alterations in the schizophrenic patients (**Figure 4A**). As shown in **Figures 5A–F**, Proteobacteria at the phylum level (VCS = 6, *p* = 0.035), and Gammaproteobacteria at the class level (VCS = 5, *p* = 0.031) were up-regulated in schizophrenic patients. At the family level, both *Enterobacteriaceae* (VCS = 6, *p* = 0.016) and *Lactobacillaceae* (VCS = 6, *p* = 0.035) were increased. At the genus level, *Lactobacillus* (VCS = 9, *p* = 0.002), *Prevotella* (VCS = 7, *p* = 0.008), *Succinivibrio* (VCS =7, *p* = 0.008) and *Acidaminococcus* (VCS = 6, *p* = 0.016) were enriched, whereas *Fusicatenibacter* (VCS = -9, *p* = 0.002), *Roseburia* (VCS = -7, *p* = 0.008), *Coprococcus* (VCS = -7, *p* = 0.008), *Faecalibacterium* (VCS = -6, *p* = 0.035) and *Anaerostipes* (VCS = -6, *p* = 0.016) were depleted in schizophrenia. No significant difference was observed for candidate microbiota at order and species levels.

### 3.4 Effects of demographic and methodological heterogeneity on microbial alterations

In order to explore the effects of potential confounding factors on microbiota, we performed subgroup analyzes. We stratified population based on the PANSS total score at baseline for untreated patients so as to investigate the influence of disease severity (**Supplementary Table S5**). Patients with a mean PANSS total score ranging from 61 to 78 were classified as the mildly ill group (2 studies), and those with a mean PANSS total score ranging from 78 to 96 were classified as the moderately ill group (8 studies) (38). However, we only compared the moderately ill group with the entire patient population due to the limited studies for the mildly ill group. The number of candidate taxa for the moderately ill group decreased by 68 (**Supplementary Figure S2**; **Supplementary Table S7**). The result of vote counting analysis showed that only *Fusicatenibacter* remained dysregulated in the moderately diseased patients (VCS = -6, *p* = 0.016, **Figure 4B**; **Supplementary Table S8**).

Further, we explored the effect of sequencing method, and summarized the microbial changes resulted from 23 studies using 16S rRNA amplicon sequencing, since the data for studies using metagenomic sequencing are limited (6 studies). We found that the number of candidate microbiota obtained from 16S rRNA amplicon sequencing decreased by 13 (**Supplementary Figure S3**; **Supplementary Table S7**). Notably, *Bacteroides* extraly exhibited significant difference (VCS = -6, *p* = 0.035), while *Faecalibacterium* (VCS = -5, *p* = 0.063) no longer demonstrated statistical significance comparing to the results from the entire datasets (**Figure 4C**; **Supplementary Table S8**).

To investigate the influence of drug treatments, we compared the microbiota results between the untreated patients (30 studies) and entire patient group since the data for drug treated group are limited (6 studies). We observed the number of candidate microbiota at the genus level decreased by 8 in the untreated group (**Supplementary Figure S4**; **Supplementary Table S7**). Additionally, *Ruminococcus* showed an decrease in untreated patients comparing to the results from the entire group (VCS = -6, *p* = 0.016, **Figure 4D**; **Supplementary Table S8**).

Different geographic locations may lead to the variation of gut microbiome attributed to different dietary patterns. Most included studies (22/30) were performed in China, so we compared the gut microbiota between Chinese patients and the entire patient group. The number of candidate microbiota within the Chinese group reduced by 9 (**Supplementary Figure S5**; **Supplementary Table S7**). *Lactobacillaceae* no longer demonstrated an increase in Chinese patients (VCS = 4, *p* = 0.109, **Figure 4E**; **Supplementary Table S8**).

To further explore the association between age and gut microbiome, we classified Chinese patients into the youth group (18≤Mean Age<35), and the middle and elderly group (Mean Age≥35) based on the mean age reported in each study (**Supplementary Table S5**) (39, 40). The youth group (10 studies) and middle and elderly group (12 studies) exhibited a total of 24 and 40 candidate microbiota, respectively (**Supplementary Figures S6**, **S7**; **Supplementary Table S7**). Lower level of *Blautia* was observed in the youth group, which was not found in all patients (VCS = -5, *p* = 0.031, **Figure 4F**; **Supplementary Table S8**). In the middle and elderly group, we observed increased levels of Proteobacteria (VCS = 5, *p* = 0.031), *Succinivibrionaceae* (VCS = 5, *p* = 0.031), *Succinivibrio* (VCS = 7, *p* = 0.008), *Prevotella* (VCS = 6, *p* = 0.016), *Acidaminococcus*(VCS = 5, *p* = 0.031), *Lactobacillus* (VCS = 5, *p* = 0.031) and *Megasphaera* (VCS = 5, *p* = 0.031), and decreased levels of *Roseburia* (VCS = -5, *p* = 0.031), *Faecalibacterium* (VC S = -5, *p* = 0.031), and *Coprococcus* (VCS = -5, *p* = 0.031) (**Figure 4G**; **Supplementary Table S8**). Among these microbiota, differential *Succinivibrionaceae* and *Megasphaera* were not revealed in the entire patient group.

## 4 Discussion

This study is the first comprehensive investigation of the gut microbiota changes in patients with schizophrenia, based on 428 differential gut microbial taxa from 30 studies. We assessed the reproducibility and stability of potential gut microbiota markers by vote counting analysis. Ultimately, we identified a total of 13 gut microbial taxa that showed consistent disturbance in the schizophrenic patients.

Highly diverse gut microbiota is commonly considered to be important for host health (41, 42). This assumption seems to be consistent with other research which found a reduction in the diversity index among untreated or medicated patients with schizophrenia (21, 43, 44). Surprisingly, we found no strong evidence for a significant difference in α-diversity of microbiota between the schizophrenia and control groups after analyzing our synthesized data. Simultaneously, despite more than half of the findings indicating significant differences in β-diversity between the schizophrenia and control groups, the meaning of these disparities remains uncertain.

We observed consistent down-regulation of *Roseburia*, *Faecalibacterium*, *Coprococcus*, *Anaerostipes* and *Fusicatenibacter* at genus level in patients with schizophrenia. The shared feature among these genera lies in their capacity to synthesize short-chain fatty acids (SCFAs) (45–49). The reduction of these taxa can result in inadequate production of SCFAs in the intestine. SCFAs, which are crucial end products resulting from bacterial fermentation in the intestinal tract, are believed to exert a protective effect against intestinal inflammation through various mechanisms (50, 51). For example, SCFAs have been reported to exert inhibitory effects on the production of pro-inflammatory cytokines, enhance the expression of IL-10, activate regulatory T-cell (Tregs), in turn reduce colon inflammation (52). Alternatively, intestinal epithelial cell-mediated SCFAs binds to the G protein-coupled receptor (GPR43) and activates the inflammasome-activating protein NLRP3, inducing the release of IL-18, which has a protective effect on enteritis (53, 54). Moreover, butyric acid, one of SCFAs, not only provides energy for intestinal epithelial cells but also strengthens the intestinal defense barrier to protect against intestinal inflammation (55, 56). It is now widely accepted that neuroinflammation play important roles in the pathogenesis of schizophrenia (57–59). Inflammatory factors can produce in the intestinal inflammation, circulates through the bloodstream, and induce damage to the blood-brain barrier or enter the brain following interaction with astrocytes (60, 61). Activation of microglia results in an inflammatory response in the central nervous system, thereby increasing the disease risk to schizophrenia (20, 62, 63). These mechanisms also support the findings of previous studies that have reported a higher incidence of intestinal inflammation in individuals diagnosed with schizophrenia (20, 63).

We also found consistently up-regulated of *Prevotella* and *Lactobacillus* in patients with schizophrenia at genus level. Previous studies have demonstrated that *Prevotella* at high abundance levels disrupts intestinal immune function by producing IgA proteases, thereby having negative consequences for brain (64). *Lactobacillus* is one of the most well recognized probiotics nowadays (65). However, the abnormal up-regulation of *Lactobacillus* abundance can lead to excessive production of lactate, resulting in the accumulation of lactate in the brain. This disrupts mitochondrial energy regulation balance and triggers a series of mental disorders (66). Additionally, it was found that the abundance level of *Lactobacillus* is negatively correlated with the concentration of butyric acid in SCFAs (67).

Notably, although we found a consistently up-regulated trend of Proteobacteria (phylum), Gammaproteobacteria (class), *Lactobacillaceae* (family), *Enterobacteriaceae* (family), *Succinivibrio* (genus) and *Acidaminococcus* (genus) in our study, it is difficult to accurately explore their relationship with schizophrenia due to the higher taxa or limited reports. Nevertheless, our research results provide valuable insights for further investigation into the role of dysbiosis of these microbiota in schizophrenia mechanisms.

Although it is unrealistic to have complete control over confounding factors, we can selectively control significant confounding factors that are relevant to the research needs. After further conducting subgroup analyzes, we additionally discovered five microbiota exhibiting consistent disorder. This implies that the measurement results of these microbiota may be influenced by potential confounding factors. In the youth group, we found a consistent down-regulation of *Blautia*, which has been associated with higher aggression in other studies on patients with schizophrenia (68). This finding is particularly relevant as early-onset schizophrenia patients tended to exhibit more aggressive behavior (69). In the middle and elderly group, we observed the consistent up-regulation of *Succinivibrionaceae* (family) and *Megasphaera* (genus). This phenomenon has also been considered as being associated with aging in other studies (70, 71). A study observed a reverse up-regulation of *Ruminococcus* (genus) abundance in schizophrenia patients after medication (72). This observation coincide with what we found only in the untreated group. Due to insufficient data, this study cannot draw persuasive conclusions to demonstrate the impact of sequencing methods on microbial detection results. Additionally, considering the results of vote counting method are influenced by the amount of data and the limited amount of data collected in this study, we find it difficult to analyze the consistent dysbiosis microbiota loss exhibited by each subgroup compared to the entire patients group. This is because we cannot rule out that this phenomenon is caused by insufficient data.

Our study has certain limitations. Firstly, application of the vote counting method cannot distinguish new differential microbiota other than candidate microbiota. However, considering that only a few of the included studies provided raw data, this method remains the best approach for conducting such a large-scale quantitative analysis. If in the future, with sufficient and comprehensive original data, it is advisable to use methods with greater statistical power for further research. Secondly, although we included all relevant studies up to now, there is still an issue with insufficient available data. To avoid dilution of subgroup data, we only conducted limited subgroup analyzes in this study. If more studies provide larger amounts of data in the future, other potential confounding factors should be explored and even subgroup analyzes under multiple factors controls should be conducted. Finally, due to using a vote counting method in this study, publication bias was not examined as it is done in other ordinary meta-analyzes.

## 5 Conclusion

In conclusion, our study indicated that there was no strong evidence to support significant differences in α-diversity and β-diversity between schizophrenic patients and healthy controls. Among 428 differential gut microbial taxa collected from original studies, we found that 13 taxa consistently altered in schizophrenic patients, which was characterized by the depletion of anti-inflammatory butyrate-producing genera, and the enrichment of certain opportunistic bacteria genera and probiotics. Furthermore, the heterogeneity of schizophrenic patients had a significant impact on the measurement results of gut microbiota. Our research identified the consistent dysbiosis of microbiota in patients and analyze the impact of various confounding factors, which contributes to developing microbiota-based diagnosis and therapy for schizophrenia guided.

## Data availability statement

The raw data supporting the conclusions of this article will be made available by the authors, without undue reservation.

## Author contributions

ZL: Validation, Writing – original draft, Conceptualization. XT: Validation, Writing – review & editing. DW: Validation, Writing – review & editing. JP: Software, Writing – review & editing. YL: Software, Writing – review & editing. SG: Software, Writing – review & editing. XZ: Data curation, Writing – review & editing. DY: Writing – review & editing, Data curation. HZ: Writing – review & editing, Data curation. WT: Data curation, Writing – review & editing. WC: Writing – review & editing, Visualization. XPC: Writing – review & editing, Visualization. YC: Writing – review & editing, Visualization. XC: Writing – review & editing, Visualization. PX: Supervision, Conceptualization, Writing – review & editing.

## References

[1] Andreasen NC. Symptoms, signs, and diagnosis of schizophrenia. Lancet. (1995) 346:477–81. doi: 10.1016/S0140-6736(95)91325-4 7637483

[2] Jauhar S Johnstone M McKenna PJ. Schizophrenia. Lancet. (2022) 399:473–86. doi: 10.1016/S0140-6736(21)01730-X 35093231

[3] Kahn RS Sommer IE Murray RM Meyer-Lindenberg A Weinberger DR Cannon TD. Schizophrenia. Nat Rev Dis Primers. (2015) 1:15067. doi: 10.1038/nrdp.2015.67 27189524

[4] Long J Huang G Liang W Liang B Chen Q Xie J. The prevalence of schizophrenia in mainland China: evidence from epidemiological surveys. Acta Psychiatr Scand. (2014) 130:244–56. doi: 10.1111/acps.12296 24916190

[5] McGrath J Saha S and Chant D Welham J. Schizophrenia: a concise overview of incidence, prevalence, and mortality. Epidemiol Rev. (2008) 30:67–76. doi: 10.1093/epirev/mxn001 18480098

[6] Laursen TM Nordentoft M Mortensen PB. Excess early mortality in schizophrenia. Annu Rev Clin Psychol. (2014) 10:425–48. doi: 10.1146/annurev-clinpsy-032813-153657 24313570

[7] Farmer AE McGuffin P. The pathogenesis and management of schizophrenia. Drugs. (1988) 35:177–85. doi: 10.2165/00003495-198835020-00006 2895702

[8] Chan MK Cooper JD Bahn S. Commercialisation of biomarker tests for mental illnesses: advances and obstacles. Trends Biotechnol. (2015) 33:712–23. doi: 10.1016/j.tibtech.2015.09.010 26549771

[9] Stępnicki P Kondej M Kaczor AA. Current concepts and treatments of schizophrenia. Molecules. (2018) 23(8):2087. doi: 10.3390/molecules23082087 PMC6222385 30127324

[10] Long-Smith C O'Riordan KJ Clarke G Stanton C Dinan TG Cryan JF. Microbiota-gut-brain axis: new therapeutic opportunities. Annu Rev Pharmacol Toxicol. (2020) 60:477–502. doi: 10.1146/annurev-pharmtox-010919-023628 31506009

[11] The Human Microbiome Project Consortium. Structure, function and diversity of the healthy human microbiome. Nature. (2012) 486:207–14. doi: 10.1038/nature11234 PMC3564958 22699609

[12] Golofast B Vales K. The connection between microbiome and schizophrenia. Neurosci Biobehav Rev. (2020) 108:712–31. doi: 10.1016/j.neubiorev.2019.12.011 31821833

[13] Kelly JR Minuto C Cryan JF Clarke G Dinan TG. The role of the gut microbiome in the development of schizophrenia. Schizophr Res. (2021) 234:4–23. doi: 10.1016/j.schres.2020.02.010 32336581

[14] Rhee SH Pothoulakis C Mayer EA. Principles and clinical implications of the brain-gut-enteric microbiota axis. Nat Rev Gastroenterol Hepatol. (2009) 6:306–14. doi: 10.1038/nrgastro.2009.35 PMC3817714 19404271

[15] Collins SM Kassam Z Bercik P. The adoptive transfer of behavioral phenotype via the intestinal microbiota: experimental evidence and clinical implications. Curr Opin Microbiol. (2013) 16:240–5. doi: 10.1016/j.mib.2013.06.004 23845749

[16] Socała K Doboszewska U Szopa A Serefko A Włodarczyk M Zielińska A. The role of microbiota-gut-brain axis in neuropsychiatric and neurological disorders. Pharmacol Res. (2021) 172:105840. doi: 10.1016/j.phrs.2021.105840 34450312

[17] Shen Y Xu J Li Z Huang Y Yuan Y Wang J. Analysis of gut microbiota diversity and auxiliary diagnosis as a biomarker in patients with schizophrenia: A cross-sectional study. Schizophr Res. (2018) 197:470–7. doi: 10.1016/j.schres.2018.01.002 29352709

[18] Agus A Planchais J Sokol H. Gut microbiota regulation of tryptophan metabolism in health and disease. Cell Host Microbe. (2018) 23:716–24. doi: 10.1016/j.chom.2018.05.003 29902437

[19] Morris G Berk M Carvalho A Caso JR Sanz Y Walder K. The role of the microbial metabolites including tryptophan catabolites and short chain fatty acids in the pathophysiology of immune-inflammatory and neuroimmune disease. Mol Neurobiol. (2017) 54:4432–51. doi: 10.1007/s12035-016-0004-2 27349436

[20] Birnbaum R Jaffe AE Chen Q Shin JH Kleinman JE Hyde TM. Investigating the neuroimmunogenic architecture of schizophrenia. Mol Psychiatry. (2018) 23:1251–60. doi: 10.1038/mp.2017.89 28485405

[21] Zheng P Zeng B Liu M Chen J Pan J Han Y. The gut microbiome from patients with schizophrenia modulates the glutamate-glutamine-GABA cycle and schizophrenia-relevant behaviors in mice. Sci Adv. (2019) 5:eaau8317. doi: 10.1126/sciadv.aau8317 30775438 PMC6365110

[22] Zhu F Ju Y Wang W Wang Q Guo R Ma Q. Metagenome-wide association of gut microbiome features for schizophrenia. Nat Commun. (2020) 11:1612. doi: 10.1038/s41467-020-15457-9 32235826 PMC7109134

[23] Zhu F Guo R Wang W Ju Y Wang Q Ma Q. Transplantation of microbiota from drug-free patients with schizophrenia causes schizophrenia-like abnormal behaviors and dysregulated kynurenine metabolism in mice. Mol Psychiatry. (2020) 25:2905–18. doi: 10.1038/s41380-019-0475-4 31391545

[24] Nikolova VL Smith MRB Hall LJ Cleare AJ Stone JM Young AH. Perturbations in gut microbiota composition in psychiatric disorders: A review and meta-analysis. JAMA Psychiatry. (2021) 78:1343–54. doi: 10.1001/jamapsychiatry.2021.2573 PMC8444066 34524405

[25] Järbrink-Sehgal E Andreasson A. The gut microbiota and mental health in adults. Curr Opin Neurobiol. (2020) 62:102–14. doi: 10.1016/j.conb.2020.01.016 32163822

[26] Schwarz E Maukonen J Hyytiäinen T Kieseppä T Orešič M Sabunciyan S. Analysis of microbiota in first episode psychosis identifies preliminary associations with symptom severity and treatment response. Schizophr Res. (2018) 192:398–403. doi: 10.1016/j.schres.2017.04.017 28442250

[27] Ling Z Liu X Cheng Y Yan X Wu S. Gut microbiota and aging. Crit Rev Food Sci Nutr. (2022) 62:3509–34. doi: 10.1080/10408398.2020.1867054 33377391

[28] Yuan X Wang Y Li X Jiang J Kang Y Pang L. Gut microbial biomarkers for the treatment response in first-episode, drug-naïve schizophrenia: a 24-week follow-up study. Transl Psychiatry. (2021) 11:422. doi: 10.1038/s41398-021-01531-3 34376634 PMC8355081

[29] Cuervo A Valdés L Salazar N Reyes-Gavilán los CG Ruas-Madiedo P Gueimonde M. Pilot study of diet and microbiota: interactive associations of fibers and polyphenols with human intestinal bacteria. J Agric Food Chem. (2014) 62:5330–6. doi: 10.1021/jf501546a 24877654

[30] Cheniaux E Landeira-Fernandez J Versiani M. The diagnoses of schizophrenia, schizoaffective disorder, bipolar disorder and unipolar depression: interrater reliability and congruence between DSM-IV and ICD-10. Psychopathology. (2009) 42:293–8. doi: 10.1159/000228838 19609099

[31] Quinones MP Kaddurah-Daouk R. Metabolomics tools for identifying biomarkers for neuropsychiatric diseases. Neurobiol Dis. (2009) 35:165–76. doi: 10.1016/j.nbd.2009.02.019 19303440

[32] Naseribafrouei A Hestad K Avershina E Sekelja M Linløkken A Wilson R. Correlation between the human fecal microbiota and depression. Neurogastroenterol Motil. (2014) 26:1155–62. doi: 10.1111/nmo.12378 24888394

[33] Page MJ McKenzie JE Bossuyt PM Boutron I Hoffmann TC Mulrow CD. The PRISMA 2020 statement: an updated guideline for reporting systematic reviews. BMJ. (2021) 372:n71. doi: 10.1136/bmj.n71 33782057 PMC8005924

[34] Pizzol D Demurtas J Celotto S Maggi S Smith L Angiolelli G. Urinary incontinence and quality of life: a systematic review and meta-analysis. Aging Clin Exp Res. (2021) 33:25–35. doi: 10.1007/s40520-020-01712-y 32964401 PMC7897623

[35] Rikke BA Wynes MW Rozeboom LM Barón AE Hirsch FR. Independent validation test of the vote-counting strategy used to rank biomarkers from published studies. biomark Med. (2015) 9:751–61. doi: 10.2217/BMM.15.39 PMC4770796 26223535

[36] Goveia J Pircher A Conradi LC Kalucka J Lagani V Dewerchin M. Meta-analysis of clinical metabolic profiling studies in cancer: challenges and opportunities. EMBO Mol Med. (2016) 8:1134–42. doi: 10.15252/emmm.201606798 PMC5048364 27601137

[37] Ling Z Jin G Yan X Cheng Y Shao L Song Q. Fecal dysbiosis and immune dysfunction in chinese elderly patients with schizophrenia: an observational study. Front Cell Infect Microbiol. (2022) 12:886872. doi: 10.3389/fcimb.2022.886872 35719348 PMC9198589

[38] Leucht S Kane JM Kissling W Hamann J Etschel E Engel RR. What does the PANSS mean? Schizophr Res. (2005) 79:231–8. doi: 10.1016/j.schres.2005.04.008 15982856

[39] Kolenic M Franke K Hlinka J Matejka M Capkova J Pausova Z. Obesity, dyslipidemia and brain age in first-episode psychosis. J Psychiatr Res. (2018) 99:151–8. doi: 10.1016/j.jpsychires.2018.02.012 29454222

[40] Croca M Lagodka A Gadel R Bourdel MC Bendjemaa N Gaillard R. Theory of mind and schizophrenia in young and middle-aged patients: Influence of executive functions. Psychiatry Res. (2018) 259:532–7. doi: 10.1016/j.psychres.2017.10.041 29156426

[41] Shade A. Diversity is the question, not the answer. Isme J. (2017) 11:1–6. doi: 10.1038/ismej.2016.118 27636395 PMC5421358

[42] Gerritsen J Smidt H Rijkers GT de Vos WM. Intestinal microbiota in human health and disease: the impact of probiotics. Genes Nutr. (2011) 6:209–40. doi: 10.1007/s12263-011-0229-7 PMC3145058 21617937

[43] Xu R Wu B Liang J He F Gu W Li K. Altered gut microbiota and mucosal immunity in patients with schizophrenia. Brain Behav Immun. (2020) 85:120–7. doi: 10.1016/j.bbi.2019.06.039 31255682

[44] Ma X Asif H Dai L He Y Zheng W Wang D. Alteration of the gut microbiome in first-episode drug-naïve and chronic medicated schizophrenia correlate with regional brain volumes. J Psychiatr Res. (2020) 123:136–44. doi: 10.1016/j.jpsychires.2020.02.005 32065949

[45] Calderón-Pérez L Gosalbes MJ Yuste S Valls RM Pedret A Llauradó E. Gut metagenomic and short chain fatty acids signature in hypertension: a cross-sectional study. Sci Rep. (2020) 10:6436. doi: 10.1038/s41598-020-63475-w 32296109 PMC7160119

[46] van den Munckhof ICL Kurilshikov A Horst Ter R Riksen NP Joosten LAB Zhernakova A. Role of gut microbiota in chronic low-grade inflammation as potential driver for atherosclerotic cardiovascular disease: a systematic review of human studies. Obes Rev. (2018) 19:1719–34. doi: 10.1111/obr.12750 30144260

[47] Zhang W Xu JH Yu T Chen QK. Effects of berberine and metformin on intestinal inflammation and gut microbiome composition in db/db mice. BioMed Pharmacother. (2019) 118:109131. doi: 10.1016/j.biopha.2019.109131 31545226

[48] Lo Presti A Zorzi F Chierico Del F Altomare A Cocca S Avola A. Fecal and mucosal microbiota profiling in irritable bowel syndrome and inflammatory bowel disease. Front Microbiol. (2019) 10:1655. doi: 10.3389/fmicb.2019.01655 31379797 PMC6650632

[49] Zhou J Wu X Li Z Zou Z Dou S Li G. Alterations in gut microbiota are correlated with serum metabolites in patients with insomnia disorder. Front Cell Infect Microbiol. (2022) 12:722662. doi: 10.3389/fcimb.2022.722662 35252021 PMC8892143

[50] Morrison DJ Preston T. Formation of short chain fatty acids by the gut microbiota and their impact on human metabolism. Gut Microbes. (2016) 7:189–200. doi: 10.1080/19490976.2015.1134082 26963409 PMC4939913

[51] Martin-Gallausiaux C Marinelli L Blottière HM Larraufie P Lapaque N. SCFA: mechanisms and functional importance in the gut. Proc Nutr Soc. (2021) 80:37–49. doi: 10.1017/S0029665120006916 32238208

[52] Smith PM Howitt MR Panikov N Michaud M Gallini CA Bohlooly YM. The microbial metabolites, short-chain fatty acids, regulate colonic Treg cell homeostasis. Science. (2013) 341:569–73. doi: 10.1126/science.1241165 PMC3807819 23828891

[53] Maslowski KM Vieira AT Ng A Kranich J Sierro F Yu D. Regulation of inflammatory responses by gut microbiota and chemoattractant receptor GPR43. Nature. (2009) 461:1282–6. doi: 10.1038/nature08530 PMC3256734 19865172

[54] Macia L Tan J Vieira AT Leach K Stanley D Luong S. Metabolite-sensing receptors GPR43 and GPR109A facilitate dietary fibre-induced gut homeostasis through regulation of the inflammasome. Nat Commun. (2015) 6:6734. doi: 10.1038/ncomms7734 25828455

[55] Peng L Li ZR Green RS Holzman IR Lin J. Butyrate enhances the intestinal barrier by facilitating tight junction assembly via activation of AMP-activated protein kinase in Caco-2 cell monolayers. J Nutr. (2009) 139:1619–25. doi: 10.3945/jn.109.104638 PMC2728689 19625695

[56] Huang Y Shi X Li Z Shen Y Shi X Wang L. Possible association of Firmicutes in the gut microbiota of patients with major depressive disorder. Neuropsychiatr Dis Treat. (2018) 14:3329–37. doi: 10.2147/NDT PMC6284853 30584306

[57] Na KS Jung HY Kim YK. The role of pro-inflammatory cytokines in the neuroinflammation and neurogenesis of schizophrenia. Prog Neuropsychopharmacol Biol Psychiatry. (2014) 48:277–86. doi: 10.1016/j.pnpbp.2012.10.022 23123365

[58] Ribeiro-Santos A Lucio Teixeira A Salgado JV. Evidence for an immune role on cognition in schizophrenia: a systematic review. Curr Neuropharmacol. (2014) 12:273–80. doi: 10.2174/1570159X1203140511160832 PMC4023457 24851091

[59] Severance EG Alaedini A Yang S Halling M Gressitt KL Stallings CR. Gastrointestinal inflammation and associated immune activation in schizophrenia. Schizophr Res. (2012) 138:48–53. doi: 10.1016/j.schres.2012.02.025 22446142 PMC4244845

[60] Banks WA Kastin AJ Broadwell RD. Passage of cytokines across the blood-brain barrier. Neuroimmunomodulation. (1995) 2:241–8. doi: 10.1159/000097202 8963753

[61] Verkhratsky A Steardo L Peng L Parpura V. Astroglia, glutamatergic transmission and psychiatric diseases. Adv Neurobiol. (2016) 13:307–26. doi: 10.1007/978-3-319-45096-4_12 27885635

[62] Norden DM Trojanowski PJ Villanueva E Navarro E Godbout JP. Sequential activation of microglia and astrocyte cytokine expression precedes increased Iba-1 or GFAP immunoreactivity following systemic immune challenge. Glia. (2016) 64:300–16. doi: 10.1002/glia.22930 PMC4707977 26470014

[63] Braniste V Al-Asmakh M Kowal C Anuar F Abbaspour A Tóth M. The gut microbiota influences blood-brain barrier permeability in mice. Sci Transl Med. (2014) 6:263ra158. doi: 10.1126/scitranslmed.3009759 PMC4396848 25411471

[64] Severance EG Prandovszky E Castiglione J Yolken RH. Gastroenterology issues in schizophrenia: why the gut matters. Curr Psychiatry Rep. (2015) 17:27. doi: 10.1007/s11920-015-0574-0 25773227 PMC4437570

[65] Montalban-Arques A Scharl M. Intestinal microbiota and colorectal carcinoma: Implications for pathogenesis, diagnosis, and therapy. EBioMedicine. (2019) 48:648–55. doi: 10.1016/j.ebiom.2019.09.050 PMC6838386 31631043

[66] Li H Li H Zhu Z Xiong X Huang Y Feng Y. Association of serum homocysteine levels with intestinal flora and cognitive function in schizophrenia. J Psychiatr Res. (2023) 159:258–65. doi: 10.1016/j.jpsychires.2023.01.045 36773527

[67] Wang B Kong Q Li X Zhao J Zhang H Chen W. A high-fat diet increases gut microbiota biodiversity and energy expenditure due to nutrient difference. Nutrients. (2020) 12(10):3197. doi: 10.3390/nu12103197 PMC7589760 33092019

[68] Deng H He L Wang C Zhang T Guo H Zhang H. Altered gut microbiota and its metabolites correlate with plasma cytokines in schizophrenia inpatients with aggression. BMC Psychiatry. (2022) 22:629. doi: 10.1186/s12888-022-04255-w 36167540 PMC9513873

[69] Sandyk R. Aggressive behavior in schizophrenia: relationship to age of onset and cortical atrophy. Int J Neurosci. (1993) 68:1–10. doi: 10.3109/00207459308994254 8063505

[70] Duan J Yin B Li W Chai T Liang W Huang Y. Age-related changes in microbial composition and function in cynomolgus macaques. Aging (Albany NY). (2019) 11:12080–96. doi: 10.18632/aging.v11i24 PMC6949106 31837260

[71] Takagi T Naito Y Inoue R Kashiwagi S Uchiyama K Mizushima K. Differences in gut microbiota associated with age, sex, and stool consistency in healthy Japanese subjects. J Gastroenterol. (2019) 54:53–63. doi: 10.1007/s00535-018-1488-5 29926167

[72] Gokulakrishnan K Nikhil J Viswanath B Thirumoorthy C Narasimhan S Devarajan B. Comparison of gut microbiome profile in patients with schizophrenia and healthy controls - A plausible non-invasive biomarker? J Psychiatr Res. (2023) 162:140–9. doi: 10.1016/j.jpsychires.2023.05.021 37156128

