# Supplementary material for: Alterations of the gut microbiota in patients with schizophrenia
Source: Front Psychiatry. 2024 Mar 26;15:1366311. doi: 10.3389/fpsyt.2024.1366311 (PMC11002218; doi:10.3389/fpsyt.2024.1366311)
Supplement: Supplementary file 1 [file DataSheet_1.zip › Supplementary Tables and Figures.DOCX]

**Alterations of the** **Gut Microbiota in Patients with Schizophrenia**

Zhuocan Li^1^, Xiangkun Tao^1^, Dongfang Wang^1,2,3^, Juncai Pu^1,2,4^, Yiyun Liu^1,2,3^, Siwen Gui^1,2,3^, Xiaogang Zhong^1,2,5^, Dan Yang^1^, Haipeng Zhou^1^, Wei Tao^1^, Weiyi Chen^1,4^, Xiaopeng Chen^1,4^, Yue Chen^1,4^, Xiang Chen^1,4^, Peng Xie^1,2,3,4^*

**Table S1.** PICO table.

**Table S2.** Search strategy for databases.

**Table S3.** Exclusion reasons for full‐text reports.

**Table S4.** Quality assessment of case-control studies assessed with the Newcastle Ottawa Scale.

**Table S5.** Characteristics of studies investigating gut microbiota composition in patients with schizophrenia.

**Table S6.** Microbiota diversity in patients with schizophrenia.

**Table S7.** The number of the differential gut microbiota and candidate taxa in patients with schizophrenia and subgroups.

**Table S8.** Alterations of candidate microbiota at phylum, class, order, family, genus and species levels in patients with schizophrenia and subgroups.

**Figure S1.** The lineage plot of candidate microbiota.

**Figure S2.** Bar plots of candidate microbiota for the “Moderately ill group” at phylum (A), class (B), order (C), family (D), genus (E), and species (F) levels. The vote counting statistic for each candidate microbiota is represented by orange and blue bars. An asterisk (*) indicates *p* < 0.05.

**Figure S3.** Bar plots of candidate microbiota for the “16S rRNA amplicon sequencing group” at phylum (A), class (B), order (C), family (D), genus (E), and species (F) levels. The vote counting statistic for each candidate microbiota is represented by orange and blue bars. An asterisk (*) indicates *p* < 0.05.

**Figure S4.** Bar plots of candidate microbiota for the “Untreated group” at phylum (A), class (B), order (C), family (D), genus (E), and species (F) levels. The vote counting statistic for each candidate microbiota is represented by orange and blue bars. An asterisk (*) indicates *p* < 0.05.

**Figure S5.** Bar plots of candidate microbiota for the “Chinese group” at phylum (A), class (B), order (C), family (D), genus (E), and species (F) levels. The vote counting statistic for each candidate microbiota is represented by orange and blue bars. An asterisk (*) indicates *p* < 0.05.

**Figure S6.** Bar plots of candidate microbiota for the “Youth group” at phylum (A), class (B), order (C), family (D), genus (E), and species (F) levels. The vote counting statistic for each candidate microbiota is represented by orange and blue bars. An asterisk (*) indicates *p* < 0.05.

**Figure S7.** Bar plots of candidate microbiota for the “Middle and elderly group” at phylum (A), class (B), order (C), family (D), genus (E), and species (F) levels. The vote counting statistic for each candidate microbiota is represented by orange and blue bars. An asterisk (*) indicates *p* < 0.05.

**Table S1.** PICO table.

| **Population** | **Intervention** | **Comparison** | **Outcomes** |
| --- | --- | --- | --- |
| Schizophrenia | Drug | Control | Symptom |
|  | Untreated | Placebo | Gut microbiota |
|  |  |  | Feces |
|  |  |  | 16S rRNA |
|  |  |  | Metagenome |

**Table S2.** Search strategy for databases.

| **Key terms for search of PubMed** | |
| --- | --- |
| #1 | gut microbiota[MeSH Terms] OR ((gut[Title/Abstract] OR gastrointestin*[Title/Abstract] OR intestin*[Title/Abstract] OR fecal[Title/Abstract] OR faecal[Title/Abstract] OR feces[Title/Abstract] OR stool[Title/Abstract]) AND (microbiota[Title/Abstract] OR microbiome[Title/Abstract] OR microflora[Title/Abstract] OR flora[Title/Abstract] OR bacteria[Title/Abstract] OR microbi*[Title/Abstract] OR microbe*[Title/Abstract])) OR probiotic*[Title/Abstract] OR prebiotic*[Title/Abstract] OR synbiotic*[Title/Abstract] |
| #2 | 16S rRNA[MeSH Terms] OR metagenomics[MeSH Terms] OR metagenome[MeSH Terms] OR 16S rRNA[Title/Abstract] OR 16S rDNA[Title/Abstract] OR metagenomic*[Title/Abstract] OR metagenome*[Title/Abstract] OR metaproteomic*[Title/Abstract] |
| #3 | 1 OR 2 |
| #4 | schizophrenia[MeSH Terms] OR schizophreni*[Title/Abstract] OR psychosis[Title/Abstract] OR psychotic*[Title/Abstract] |
| #5 | 3 AND 4 |
| **Key terms for search of Embase** | |
| #1 | ‘intestine flora’/exp OR ((gut OR gastrointestin* OR intestin* OR fecal OR faecal OR feces OR stool):ti,ab AND (microbiota OR microbiome OR microflora OR flora OR bacteria OR microbi* OR microbe*):ti,ab) OR (probiotic* OR prebiotic* OR synbiotic*):ti,ab |
| #2 | ‘RNA 16S’/exp OR ‘DNA 16S’/exp OR ‘metagenomics’/exp OR ‘metagenome’/exp OR ‘metaproteomics’/exp OR (‘16S rRNA’ OR ‘16S rDNA’ OR metagenomic* OR metagenome* OR metaproteomic*):ti,ab |
| #3 | #1 OR #2 |
| #4 | ‘schizophrenia’/exp OR (schizophreni* OR psychosis OR psychotic*):ti,ab |
| #5 | 3 AND 4 |
| **Key terms for search of Web of Science** | |
| #1 | (TS=(gut OR gastrointestin* OR intestin* OR fecal OR faecal OR feces OR stool)) AND TS=(microbiota OR microbiome OR microflora OR flora OR bacteria OR microbi* OR microbe*) |
| #2 | TS=(probiotic* OR prebiotic* OR synbiotic*) |
| #3 | TS=("16S rRNA" OR "16S rDNA" OR metagenomic* OR metagenome* OR metaproteomic*) |
| #4 | #1 OR #2 OR #3 |
| #5 | TS=(schizophreni* OR psychosis OR psychotic*) |
| #6 | #4 AND #5 |
| **Key terms for search of Cochrane Library** | |
| #1 | MeSH descriptor: [gastrointestinal microbiome] explode all trees |
| #2 | (gut OR gastrointestin* OR intestin* OR fecal OR faecal OR feces OR stool):ti,ab,kw AND (microbiota OR microbiome OR microflora OR flora OR bacteria OR microbi* OR microbe*):ti,ab,kw |
| #3 | (probiotic* OR prebiotic* OR synbiotic*):ti,ab,kw |
| #4 | #1 OR #2 OR #3 |
| #5 | MeSH descriptor: [RNA, ribosomal, 16S] explode all trees |
| #6 | MeSH descriptor: [metagenomics] explode all trees |
| #7 | MeSH descriptor: [metagenome] explode all trees |
| #8 | ‘16S rRNA’ OR ‘16S rDNA’ OR metagenomic* OR metagenome* OR metaproteomic* |
| #9 | #5 OR #6 OR #7 OR #8 |
| #10 | #4 OR #9 |
| #11 | MeSH descriptor: [schizophrenia] explode all trees |
| #12 | (schizophreni* OR psychosis OR psychotic*):ti,ab,kw |
| #13 | #11 OR #12 |
| #14 | #10 AND #13 |

**Table S3.** Exclusion reasons for full‐text reports.

| **Full-text excluded articles.** | | |
| --- | --- | --- |
| **Title** | **Study name** | **Excluded reasons** |
| Evaluating the Genetic Effects of Gut Microbiota on the Development of Neuroticism and General Happiness: A Polygenic Score Analysis and Interaction Study Using UK Biobank Data | Jia Y 2023 | No relevant study |
| Treatment Resistance in Schizophrenia Is Associated with Attention Deficit/Hyperactivity Disorder and Gut Microbiota: A Genetic Correlation and Mendelian Randomization Study | Cheng B 2023 | No available data |
| The antipsychotic drug olanzapine altered lipid metabolism in the common carp (Cyprinus carpio L.): Insight from the gut microbiota-SCFAs-liver axis | Chang X 2023 | No study subject of interest |
| Gut mycobiota dysbiosis in drug-naïve, first-episode schizophrenia | Yuan X 2022 | No available data |
| Mapping trends and hotspot regarding gastrointestinal microbiome and neuroscience: A bibliometric analysis of global research (2002-2022) | Yang J 2022 | No available data |
| The schizophrenia and gut microbiota: A bibliometric and visual analysis | Yang C 2022 | No available data |
| Nutritional Impact and Eating Pattern Changes in Schizophrenic Spectrum Disorders after Health Education Program on Symbiotic Dietary Modulation Offered by Specialised Psychiatric Nursing-Two-Arm Randomised Clinical Trial | Sevillano-Jiménez A 2022 | No available data |
| Impact of high prebiotic and probiotic dietary education in the SARS-CoV-2 era: improved cardio-metabolic profile in schizophrenia spectrum disorders | Sevillano-Jiménez A 2022 | No available data |
| Efficacy of nutrition education for the increase of symbiotic intake on nutritional and metabolic status in schizophrenic spectrum disorders: A two-arm protocol | Sevillano-Jiménez A 2022 | Other types of reports (review, case report, protocol, commentary, editorial) |
| Targeted metabolomics reveals aberrant profiles of serum bile acids in patients with schizophrenia | Qing Y 2022 | No available data |
| Antibiotics induced gut microbiota disbiosis rescues the behavioral phenotype in a maternal immune-activation (MIA) model of autism spectrum disorder / schizophrenia | Pacheco-LГіpez G 2022 | Others |
| Gut Microbiota and Psychiatric Disorders: A Two-Sample Mendelian Randomization Study | Ni J 2021 | No relevant study |
| Prebiotic Treatment in People With Schizophrenia | - | Others |
| Investigating the interaction between the human gut microbiome and clozapine therapy in schizophrenia | Liu JCW 2022 | Others |
| Gut dysbiosis impairs hippocampal plasticity and behaviors by remodeling serum metabolome | Liu G 2022 | No relevant study |
| Enhanced intestinal protein fermentation in schizophrenia | Liang Y 2022 | No relevant study |
| An integrative study of the microbiome gut-brain-axis and hippocampal inflammation in psychosis: Persistent effects from mode of birth | Joe P 2021 | No available data |
| Evaluation of the effect of probiotic supplements as adjunctive therapy in improving the symptoms of psychosis, anxiety, insomnia and anorexia due to amphetamine and methamphetamine use | - | Others |
| Use of probiotics in the treatment of schizophrenia | - | Others |
| Global metabolic profiles in a non-human primate model of maternal immune activation: implications for neurodevelopmental disorders | Boktor JC 2022 | No relevant study |
| Bacterial Translocation Associates With Aggression in Schizophrenia Inpatients | Wang C 2021 | No relevant study |
| Effects of Berberine on Gut Microbiota in Patients with Mild Metabolic Disorders Induced by Olanzapine | Pu Z 2021 | No relevant study |
| No Guts No Glory dietary intervention | - | Others |
| Study Protocol: The Evaluation Study for Social Cognition Measures in Japan (ESCoM) | - | Other types of reports (review, case report, protocol, commentary, editorial) |
| Prebiotic Treatment Increases Serum Butyrate in People With Schizophrenia: Results of an Open-Label Inpatient Pilot Clinical Trial | Kelly DL 2021 | Other types of reports (review, case report, protocol, commentary, editorial) |
| Probiotic supplements reduce antipsychotic-induced metabolic disturbances in drug-naive first-episode schizophrenia | Kang D 2021 | Others |
| Evaluation of the Effect of BioZen D as Adjunctive therapy in improving Positive, Negative, Cognitive symptoms and Risk factors for Metabolic syndrome and Inflammatory markers in Schizophrenia patients | - | Others |
| S. P.0480 Patients with schizophrenia have significantly different stool bacteriome | FliegerovГЎ KO 2021 | Others |
| COMPARISON OF THE COMBINED TREATMENT OF THE LIPOSOMATED POLYPHENOLS CГљRCULA AND RESVERATROLВ® WITH DUTASTERIDE, IN THE IMPROVEMENT OF PATIENTS WITH ELA WHO ARE BEING TREATED WITH RILUZOLE | - | Others |
| A Randomized Clinical Trial of Fecal Microbiota Transplant for Treatment-Resistant Schizophrenia | - | Others |
| Efficacy of Prebiotic and Probiotic Dietary Modulation in Schizophrenic Disorders | - | Others |
| Synbiotic Compound to Reduce Symptoms of Schizophrenia | - | Others |
| Antipsychotic Effects of Probiotics and Prebiotics on Patients With Schizophrenia | - | Others |
| Gene-Environment Interactions in Major Mental Disorders in the Czech Republic | Latalova K 2020 | No relevant study |
| The effects of prebiotic treatment in schizophrenia: Results of a pilot study | Kelly D 2021 | Meeting abstracts |
| A discriminative analysis of schizophrenia using EEG signal combined with intestinal microbiota | Jiahui L 2020 | Others |
| Clinical Trials of Immuno-Modulatory Therapies for Serious Mental Illness | Dickerson F 2020 | Others |
| A 12-Week Randomized, Double-Blind, Placebo-Controlled Study of Baicalin in the Treatment of Drug-Induced Obesity in Schizophrenia | - | Others |
| Effect of ketogenic diet combined with olanzapine on metabolic disorder and cognitive symptoms in schizophrenia | - | Others |
| Pro-cognitive effect of a prebiotic in psychosis: A double blind placebo controlled cross-over study | Kao A 2019 | No available data |
| Clinical trial of investigation of add-on of Probiotics to standard treatment on symptoms of patients with first episode schizophrenia | - | Others |
| Effect of Synbiotic Supplementation on schizophrenia symptoms | - | Others |
| Consequences of gut microbiota manipulation by antibiotics in animal model of early immune dysregulation: Phenotype analysis of behaviour and biochemical correlates | TejkalovГЎ H 2018 | Others |
| Documenting and treating a dysregulated gutimmune-brain axis in schizophrenia | Severance EG 2018 | Others |
| Animal models of mood disorders and the evaluation of probiotics | Misheneva V 2018 | Others |
| Effect of probiotic and selenium co-supplementation in the treatment of schizophrenia patients | - | Others |
| Gut microbial community structure varies with atypical antipsy-chotic treatment and with resistant starch in a bipolar and schizophrenia cohort | Flowers S 2018 | Others |
| A study examining the effect of consuming foods containing probiotics on anxiety and depressive symptoms : a non-randomized and open trial | - | Others |
| Efficacy of Clostridium butyricum M588 probiotics for the patients of constipation in schizophrenia: 4 weeks, a randomized, double blinded trial | - | Others |
| Gender specific behavioral alterations are associated with gut dysbiosis in mice exposed to multifactorial early-life adversity | Rincel M 2017 | Others |
| The Effects of Prebiotics on Cognitive Functioning and Weight Gain in Psychosis | - | Others |
| A 8-weeks Study to Evaluate the Probiotics Treatment in Prevention and Intervention of Weight-gain and Cognitive Impairment of First Episode Schizophrenia | - | Others |
| The role of maternal gut microbiome in perinatal neurodevelopment: Implications for neurodevelopmental disorders | Lebovitz Y 2017 | Others |
| Effect of supplementation in treatment of schizophrenia patients | - | Others |
| The role of the microbiome in bipolar disorder-a new model | Dickerson F 2017 | Others |
| Intestinal inflammation, the microbiome, and human neuropsychiatric disorders | Yolken R 2016 | Others |
| Minocycline add-on to Antipsychotics for the Treatment of Negative and Cognitive Symptoms in Schizophrenia | - | Others |
| The gut-immune-brain interactome and microbial dysbiosis in psychiatric disorders | Severance EG 2015 | Others |
| Effect of probiotic supplementation on schizophrenia symptoms and association with gastrointestinal functioning: a randomized, placebo-controlled trial | Dickerson FB 2014 | No differential analysis |
| The other human genome: The role of the microbial metagenome in the pathogenesis of schizophrenia | Yolken R 2013 | Meeting abstracts |
| Gastrointestinal symptoms in a mouse model of an environmental risk factor for autism and schizophrenia | Patterson PH 2013 | Meeting abstracts |
| Differences in gut microbiome between schizophrenic patients and healthy individuals | Ang KYR 2013 | Others |
| Double-Blind Trial of a Probiotic Supplement to Reduce the Symptoms of Schizophrenia | - | No differential analysis |
| Decreased Clostridium Abundance after Electroconvulsive Therapy in the Gut Microbiota of a Patient with Schizophrenia | Kanayama M 2019 | Other types of reports (review, case report, protocol, commentary, editorial) |
| Immunomodulatory Effects of Probiotic Supplementation in Schizophrenia Patients: A Randomized, Placebo-Controlled Trial | Tomasik J 2015 | No available data |
| Ketogenic diet reverses behavioral abnormalities in an acute NMDA receptor hypofunction model of schizophrenia | Kraeute AK 2015 | Other types of reports (review, case report, protocol, commentary, editorial) |
| A gluten-free diet in people with schizophrenia and anti-tissue transglutaminase or anti-gliadin antibodies | Jackson J 2012 | Other types of reports (review, case report, protocol, commentary, editorial) |
| Microbiota modulate behavioral and physiological abnormalities associated with neurodevelopmental disorders | Hsiao EY 2013 | No relevant study |
| Changes in metabolism and microbiota after 24-week risperidone treatment in drug naïve, normal weight patients with first episode schizophrenia | Yuan X 2018 | Non-high throughput detection technique |
| Gut Microbiota Markers for Antipsychotics Induced Metabolic Disturbance in Drug Naïve Patients with First Episode Schizophrenia – A 24 Weeks Follow-up Study | Li X 2021 | No relevant study |
| Composition, taxonomy and functional diversity of the oropharynx microbiome in individuals with schizophrenia and controls | Castro-Nallar E 2015 | No relevant study |
| Metagenome-wide association of gut microbiome features for schizophrenia | Zhu F 2020 | No relevant study |
| In Schizophrenia, Increased Plasma IgM/IgA Responses to Gut Commensal Bacteria Are Associated with Negative Symptoms, Neurocognitive Impairments, and the Deficit Phenotype | Maes M 2018 | No available data |
| The role of the gut microbiome in the development of schizophrenia | Kelly JR 2021 | Other types of reports (review, case report, protocol, commentary, editorial) |
| Gut microbiome and magnetic resonance spectroscopy study of subjects at ultra-high risk for psychosis may support the membrane hypothesis | He Y 2018 | No relevant study |
| Microbiota-Orientated Treatments for Major Depression and Schizophrenia | Fond GB 2020 | Other types of reports (review, case report, protocol, commentary, editorial) |
| Alterations Of Glycerophospholipid And Fatty Acyl Metabolism In Multiple Brain Regions Of Schizophrenia Microbiota Recipient Mice | Liang W 2019 | Non-high throughput detection technique |
| Clinical and metabolic response to vitamin D plus probiotic in schizophrenia patients | Ghaderi A 2019 | No available data |
| Pro-cognitive effect of a prebiotic in psychosis: A double blind placebo controlled cross-over study | Kao ACC 2019 | Other types of reports (review, case report, protocol, commentary, editorial) |
| Transcriptome analysis in whole blood reveals increased microbial diversity in schizophrenia | Loohuis LMO 2018 | Non-high throughput detection technique |
| Effects of urban particulate matter on gut microbiome and partial schizophrenia-like symptoms in mice: Evidence from shotgun metagenomic and metabolomic profiling | Yi W 2023 | No relevant study |
| Altered fecal microbiota composition in individuals who abuse methamphetamine | Yang Y 2021 | No relevant study |
| Analysis of gut mycobiota in first-episode, drug-naïve Chinese patients with schizophrenia: A pilot study | Zhang X 2020 | No available data |
| Decreased Clostridium Abundance after Electroconvulsive Therapy in the Gut Microbiota of a Patient with Schizophrenia | Kanayama M 2019 | Other types of reports (review, case report, protocol, commentary, editorial) |
| Viral metagenomics in drug-naïve, first-onset schizophrenia patients with prominent negative symptoms | Canuti M 2015 | No available data |
| Reduced maternal levels of common viruses during pregnancy predict offspring psychosis: potential role of enhanced maternal immune activity? | Canuti M 2015 | No available data |
| Investigating the gut-brain axis in a neurodevelopmental rodent model of schizophrenia | Katz-Barber MW 2020 | No relevant study |
| HOST GENETICS INFLUENCES THE RELATIONSHIP BETWEEN THE GUT MICROBIOME AND PSYCHIATRIC DISORDERS | Martins-Silva T 2021 | No available data |
| Gut Microbiota Varies With Atypical Antipsychotic Treatment and Probiotics in a Bipolar and Schizophrenia Cohort | Flowers S 2019 | Duplicate reporting |
| Rat model of schizophrenia and gut microbiome | Fliegerova KO 2019 | Other types of reports (review, case report, protocol, commentary, editorial) |
| Bacterial infections among patients with psychiatric disorders: Relation with hospital stay, age, and psychiatric diagnoses | Belz M 2018 | No available data |
| Efect of Bifdobacterium on olanzapine‑induced body weight and appetite changes in patients with psychosis | Yang Y 2021 | No available data |
| Unravelling the antimicrobial action of antidepressants on gut commensal microbes | Chait YA 2020 | No relevant study |
| Schizophrenia phenomenology comprises a bifactorial general severity and a single-group factor, which are differently associated with neurotoxic immune and immune-regulatory pathways | Maes M 2019 | No available data |
| Breakdown of the Paracellular Tight and Adherens Junctions in the Gut and Blood Brain Barrier and Damage to the Vascular Barrier in Patients with Deficit Schizophrenia | Maes M 2019 | No available data |
| Upregulation of the Intestinal Paracellular Pathway with Breakdown of Tight and Adherens Junctions in Deficit Schizophrenia | Maes M 2019 | No available data |
| In Schizophrenia, Increased Plasma IgM/IgA Responses to Gut Commensal Bacteria Are Associated with Negative Symptoms, Neurocognitive Impairments, and the Deficit Phenotype | Maes M 2019 | No available data |
| The effect of 4G-ß-D-galactosylsucrose on antipsychotic related constipation | Nagamine T 2018 | No study subject of interest |
| 4(G)-beta-D-galactosylsucrose as a prebiotics may improve underweight in inpatients with schizophrenia | Nagamine T 2018 | No study subject of interest |
| Regulation of prefrontal cortex myelination by the microbiota | Hoban AE 2016 | No relevant study |
| The Influence of Diet and the Gut microbiota in Schizophrenia | Ghomi RH 2016 | Other types of reports (review, case report, protocol, commentary, editorial) |
| Mining microbes for mental health: Determining the role of microbial metabolic pathways in human brain health and disease | Spichak S 2021 | Other types of reports (review, case report, protocol, commentary, editorial) |
| Complement C4 associations with altered microbial biomarkers exemplify gene-by-environment interactions in schizophrenia | Severance EG 2021 | No available data |
| Associations between gut microbiota and Alzheimer's disease, major depressive disorder, andschizophrenia | Zhuang Z 2020 | Other types of reports (review, case report, protocol, commentary, editorial) |
| Clostridiales are predominant microbes that mediate psychiatric disorders | Li JJ 2020 | Other types of reports (review, case report, protocol, commentary, editorial) |
| Correction for the Research Article: “The gut microbiome from patients with schizophrenia modulates the glutamate-glutamine-GABA cycle and schizophrenia-relevant behaviors in mice” by P. Zheng, B. Zeng, M. Liu, J. Chen, J. Pan, Y. Han, Y. Liu, K. Cheng, C. Zhou, H. Wang, X. Zhou, S. Gui, S. W. Perry, M. Wong, J. Licinio, H. Wei, and P. Xie | P Zheng 2019 | Other types of reports (review, case report, protocol, commentary, editorial) |
| Anti-Candida albicans IgG Antibodies in Children With Autism Spectrum Disorders | Hughes HK 2018 | No available data |
| Investigation of the Gut Microbiome in Patients with Schizophrenia and Clozapine-Induced Weight Gain: Protocol and Clinical Characteristics of First Patient Cohorts | Gorbovskaya I 2020 | Other types of reports (review, case report, protocol, commentary, editorial) |
| Insights into the Role of Oral and Gut Microbiome in the Pathogenesis of Schizophrenia | Ghorbani M 2020 | Other types of reports (review, case report, protocol, commentary, editorial) |
| Gut Microbiome Alterations in Patients With Chronic Schizophrenia and Association With Clinical Characteristics | Nguyen T 2019 | Other types of reports (review, case report, protocol, commentary, editorial) |
| Alterations Of Glycerophospholipid And Fatty Acyl Metabolism In Multiple Brain Regions Of Schizophrenia Microbiota Recipient Mice | Liang W 2019 | No available data |
| Prebiotic attenuation of olanzapine-induced weight gain in rats: analysis of central and peripheral biomarkers and gut microbiota | Kao ACC 2018 | Non-high throughput detection technique |
| Manipulation of the Gut microbiota with a Prebiotic in Schizophrenia: A Double-Blinded Randomized Placebocontrolled Cross-over Study | Kao A 2018 | Other types of reports (review, case report, protocol, commentary, editorial) |
| The Gut Microbiome in Schizophrenia and Antipsychotic Induced Metabolic Dysfunction | Kanji S 2018 | Other types of reports (review, case report, protocol, commentary, editorial) |
| A Study of Altered Gene-Expression in Frontal-Cortex from Schizophrenic-Patients Using Differential Screening | Mulcrone J 1995 | No relevant study |
| Dietary fiber and probiotics for the treatment of atypical antipsychotic-induced metabolic side effects: study protocol for a randomized, double-blind, placebo-controlled trial | Liu C 2021 | No available data |
| Inflammation and Gut Microbiome in First-Episode Psychosis | Suvisaari J 2018 | No available data |
| Probiotic normalization of Candida albicans in schizophrenia: A randomized, placebo-controlled, longitudinal pilot study | Severance EG 2017 | No available data |
| Metagenomic Sequencing Indicates That the Oropharyngeal Phageome of Individuals With Schizophrenia Differs From That of Controls | Yolken RH 2015 | No available data |
| aternal immune activation alters adult behavior, intestinal integrity, gut microbiota and the gut inflammation | Li W 2021 | Non-high throughput detection technique |
| The Effects of Probiotic and Selenium Co-supplementation on Clinical and Metabolic Scales in Chronic Schizophrenia: a Randomized, Double-blind, Placebo-Controlled Trial | Jamilian H 2021 | No available data |
| DGGEDietary tryptophan depletion alters the faecal bacterial community structure of compulsive drinker rats in schedule-induced polydipsia | Merchan A 2021 | Non-high throughput detection technique |
| Prenatal stress-induced disruptions in microbial and host tryptophan metabolism and transport | Galley J 2021 | Non-high throughput detection technique |
| Identifying psychiatric disorder-associated gut microbiota using microbiota-related gene set enrichment analysis | Cheng S 2020 | Non-high throughput detection technique |
| Transcriptome analysis in whole blood reveals increased microbial diversity in schizophrenia | Loohuis LMO 2018 | No available data |
| Changes in metabolism and microbiota after 24-week risperidone treatment in drug naive, normal weight patients with first episode schizophrenia | Yuan X 2018 | Non-high throughput detection technique |

**Table S4.** Quality assessment of case-control studies assessed with the Newcastle Ottawa Scale.

| **Study, year** | **Is the case definition adequate?** | **Representativeness of the**  **cases** | **Selection of Controls** | **Definition of Controls** | **Comparability of cases and controls on the basis of the design or analysis** | **Ascertainment of exposure** | **Same method of ascertainment for cases and controls** | **Non-Response rate** | **Total** |
| --- | --- | --- | --- | --- | --- | --- | --- | --- | --- |
| Yan F 2022[1] | * | - | * | * | ** | - | * | * | 7 |
| Wang X 2022[2] | * | * | * | * | *- | * | * | * | 8 |
| O'Donnell M 2022[3] | * | - | * | * | *- | - | * | * | 6 |
| Misiak B 2022[4] | * | - | * | * | ** | - | * | * | 7 |
| Ma Q 2022[5] | * | * | * | * | *- | * | * | * | 8 |
| Ling Z 2022[6] | * | - | * | * | -- | - | * | * | 5 |
| Liang Y 2022[7] | * | - | * | * | *- | - | * | * | 6 |
| Gao Y 2022[8] | * | * | * | * | *- | * | * | * | 8 |
| Fan Y 2022[9] | * | * | * | * | *- | * | * | * | 8 |
| Chen Y 2022[10] | * | * | * | * | ** | * | * | * | 9 |
| Zhu C 2021[11] | * | - | * | * | ** | - | * | * | 7 |
| Yuan X 2021[12] | * | * | * | * | ** | * | * | * | 9 |
| Miao Y 2021[13] | * | * | * | * | ** | * | * | * | 9 |
| Manchia M 2021[14] | * | - | * | * | *- | - | * | * | 6 |
| Nguyen TT 2021[15] | * | - | * | * | ** | - | * | * | 7 |
| Li S 2021[16] | * | * | * | * | ** | * | * | * | 9 |
| Zhu F 2020[17] | * | * | * | * | *- | * | * | * | 8 |
| Zhang X 2019[18] | * | * | * | * | ** | * | * | * | 9 |
| Xu R 2020[19] | * | - | * | * | ** | - | * | * | 7 |
| Ma X 2020[20] | * | - | * | * | ** | - | * | * | 7 |
| Li S 2020[21] | * | * | * | * | ** | * | * | * | 9 |
| Zheng P 2019[22] | * | * | * | * | ** | * | * | * | 9 |
| Nguyen TT 2019[23] | * | - | * | * | ** | - | * | * | 7 |
| Shen Y 2018[24] | * | - | * | * | ** | - | * | * | 7 |
| Schwarz E 2017[25] | * | - | * | * | ** | - | * | * | 7 |
| He Y 2018[26] | * | - | * | * | *- | - | * | * | 7 |
| Wang Z 2023[27] | * | * | * | * | ** | * | * | * | 9 |
| Thirion F 2023[28] | * | - | * | * | *- | - | * | * | 6 |
| Li H 2023[29] | * | * | * | * | *- | * | * | * | 8 |
| Gokulakrishnan K 2023[30] | * | - | * | * | ** | - | * | * | 7 |

Note: A study can be awarded a maximum of one star for each numbered item within the Selection and Exposure categories. A maximum of two stars can be given for Comparability.

**Table S5.** Characteristics of studies investigating gut microbiota composition in patients with schizophrenia.

| **Study** | **Country** | **Sample size** | **Age (years)** **(mean ± SD)** | **Sex (male/female)** | **BMI** | **Drug treatment in patients** | **Diagnostic criteria** | **PANSS total score**  **(mean ± SD)** | **Sample** | **Sequencing method** | **Amplicon region** | **Microbial biomarkers  from comparisons** |
| --- | --- | --- | --- | --- | --- | --- | --- | --- | --- | --- | --- | --- |
| Yan F 2022[1] | China | SCZ (n=50) Control (n=50) | SCZ: 39.9 (NR) Control: 37.2 (NR) | SCZ: 50/0  Control: 50/0 | SCZ: 23.3 (NR) Control: 23.7 (NR) | NA | ICD-10 | - | Feces | 16S rRNA amplicon sequencing | V3-V4 | SCZ and Control |
| Wang X 2022[2] | China | First-episode SCZ (n=28) Control (n=29) | First-episode SCZ: 21.0 (5.9) Control: 23.5 (2.8) | First-episode SCZ: 15/13 Control: 13/16 | First-episode SCZ: 20.9 (2.6) Control: 21.5 (2.3) | Risperidone | DSM-IV-TR | First-episode SCZ: 83.4 (14.2) | Feces | 16S rRNA amplicon sequencing | V4 | First-episode SCZ (baseline) and Control (baseline) First-episode SCZ + 4-week risperidone and Control (baseline) |
| O'Donnell M 2022[3] | Australia | First-episode SCZ (n=5) SCZ + 1-year clozapine (n=17) Control (n=22) | First-episode SCZ: 21.8 (3.3) SCZ + 1-year clozapine: 44.2 (9.7) Control: 38.7 (14.9) | First-episode SCZ: 4/1 SCZ + 1-year clozapine: 8/9 Control: 12/10 | First-episode SCZ: 20.3 (2.8) SCZ + 1-year clozapine: 31.9 (6.9) Control: 27.8 (5.0) | Clozapine | DSM-V | - | Feces | 16S rRNA amplicon sequencing | V3-V4 | First-episode SCZ and Control SCZ + 1-year clozapine and Control |
| Misiak B 2022[4] | Poland | SCZ (n=53)  Control (n=58) | SCZ: 43.0 (13.7) Control: 44.4 (14.2) | SCZ: 22/31 Control: 21/37 | SCZ: 29.9 (5.9) Control: 26.6 (4.2) | NA | DSM-IV | - | Feces | 16S rRNA amplicon sequencing | V3-V4 | SCZ and Control |
| Ma Q 2022[5] | China | SCZ (n=76) Control (n=79) | SCZ: 27.9 (9.5) Control: 33.4 (12.5) | SCZ: 40/36 Control: 40/39 | SCZ: 20.63 (2.68) Control: 21.67 (2.65) | NA | DSM-IV | SCZ: 83.6 (16.7) | Feces | Metagenomic sequencing | - | SCZ and Control |
| Ling Z 2022[6] | China | Elderly SCZ (n=90) Control (n=71) | Elderly SCZ ＞ 62.0 Control ＞ 62.0 | - | - | NA | DSM-IV | - | Feces | 16S rRNA amplicon sequencing | V3-V4 | Elderly SCZ and Control |
| Liang Y 2022[7] | China | SCZ (n=97)  Control (n=69) | SCZ: 46.9 (12.8) Control: 46.4 (12.2) | SCZ: 43/54 Control: 33/36 | - | NA | SCID-IV-TR | - | Feces | 16S rRNA amplicon sequencing | V3-V4 | SCZ and Control |
| Gao Y 2022[8] | China | Paranoid SCZ (n=14) Undifferentiated SCZ (n=14) Control (n=11) | Paranoid SCZ: 41.6 (11.5) Undifferentiated SCZ: 45.6 (13.3) Control: 40.2 (6.3) | Paranoid SCZ: 14/0 Undifferentiated SCZ: 14/0 Control: 11/0 | Paranoid SCZ: 22.03 (4.65) Undifferentiated SCZ: 23.09 (4.83) Control: 21.96 (2.28) | NA | DSM-IV-TR | Paranoid SCZ: 82.6 (15.8) Undifferentiated SCZ: 84.1 (16.0) | Feces | 16S rRNA amplicon sequencing | - | Paranoid SCZ and Control Undifferentiated SCZ and Control |
| Fan Y 2022[9] | China | SCZ (n=63)  Control (n=57) | SCZ: 29 (NR) Control: 35 (NR) | SCZ: 34/29 Control: 14/43 | SCZ: 21.5 (NR) Control: 22.0 (NR) | NA | DSM | - | Feces | Metagenomic sequencing | - | SCZ and Control |
| Chen Y 2022[10] | China | SCZ (n=63)  Control (n=40) | SCZ: 21 (NR) Control: 21.5 (NR) | SCZ: 34/29 Control: 13/27 | SCZ: 20.97 (2.52) Control: 22.20 (2.83) | NA | DSM-V | SCZ: 83.1 (NR) | Feces | 16S rRNA amplicon sequencing | V3-V4 | SCZ and Control |
| Zhu C 2021[11] | China | Acute SCZ (n=32) Remission SCZ (n=30) Control (n=34) | Acute SCZ: 39.8 (11.7) Remission SCZ: 41.1 (11.0) Control: 42.1 (10.4) | Acute SCZ: 17/15 Remission SCZ: 16/14 Control: 16/18 | Acute SCZ: 22.3(3.4) Remission SCZ: 24.3 (4.1) Control: 24.3 (4.1) | NA | DSM-V | Acute SCZ: 29.8 (12.3) Remission SCZ: 10.3 (4.9) | Feces | 16S rRNA amplicon sequencing | V3-V4 | Acute SCZ and Control Remission SCZ and Control |
| Yuan X 2021[12] | China | SCZ on baseline (n=107) SCZ + 6-week risperidone (n=96) SCZ + 12-week risperidone (n=74) SCZ + 24-week risperidone (n=60) Control (n=107) | SCZ: 19.0 (NR) Control: 23.0 (NR) | SCZ: 51/56 Control: 37/70 | SCZ: 20.71 (NR) Control: 21.17 (NR) | Risperidone | DSM-IV | SCZ: 79.5 | Feces | 16S rRNA amplicon sequencing | V3-V4 | SCZ and Control |
| Miao Y 2021[13] | China | First-episode SCZ (n=100)  Control (n=90) | First-episode SCZ: 22.6 (8.2) Control: 23.0 (3.0) | First-episode SCZ: 41/59 Control: 32/58 | First-episode SCZ: 21.0 (3.5) Control: 21.2 (2.7) | NA | DSM-IV | First-episode SCZ: 86 (12) | Feces | 16S rRNA amplicon sequencing | V3-V4 | First-episode SCZ and Control |
| Manchia M 2021[14] | Italy | SCZ with treatment resistant (n=18) SCZ with treatment responsive (n=20) Control (n=20) | SCZ with treatment resistant: 44.0 (NR) SCZ with treatment responsive: 50.0 (NR) Control: 37.7 (NR) | SCZ with treatment resistant: 16/2 SCZ with treatment responsive: 18/2 Control: 13/7 | SCZ with treatment resistant: 27.3 (NR) SCZ with treatment responsive: 26.9 (NR) Control: 22.7 (NR) | Typical APs (first generation) Atypical APs (second generation) Aripiprazole (third generation) | DSM-IV-TR | - | Feces | 16S rRNA amplicon sequencing | V3-V4 | SCZ and Control SCZ with treatment resistant and Control SCZ with treatment responsive and Control |
| Nguyen TT 2021[15] | USA | SCZ (n=48) Control (n=48) | SCZ: 53.2 (10.3)  Control: 54.1 (12.6) | SCZ: 29/19 Control: 29/19 | SCZ: 31.8 (6.7) Control: 28.5 (5.9) | NA | DSM-IV-TR | - | Feces | 16S rRNA amplicon sequencing | V4 | SCZ and Control |
| Li S 2021[16] | China | SCZ (n=38) Control (n=38) | SCZ: 35.2 (10.7)  Control: 35.4 (11.5) | SCZ: 20/18 Control: 22/16 | SCZ: 23.70 (4.54) Control: 22.63 (2.63) | NA | DSM-IV | SCZ: 56.9 (19.5) | Feces | 16S rRNA amplicon sequencing | V4 | SCZ and Control |
| Zhu F 2020[17] | China | SCZ (n=90) Control (n=81) | SCZ: 28.5 (11.1)  Control: 32.8 (11.1) | SCZ: 46/44 Control: 41/40 | SCZ: 20.63 (2.75) Control: 21.69 (2.73) | NA | DSM-IV | SCZ: 72.8 (NR) | Feces | Metagenomic sequencing | - | SCZ and Control |
| Zhang X 2019[18] | China | SCZ (n=10) Control (n=16) | SCZ: 37.6 (7.2)  Control: 35.8 (6.8) | SCZ: 6/4 Control: 9/7 | SCZ: 23.3 (6.8) Control: 22.3 (6.5) | NA | DSM-IV | SCZ: 81.4 (11.9) | Feces | 16S rRNA amplicon sequencing | - | SCZ and Control |
| Xu R 2020[19] | China | SCZ-Metagenome (n=40) Control-Metagenome (n=40) SCZ-16S rRNA (n=44) Control-16S rRNA (n=37) | SCZ-Metagenome: 35 (11) Control-Metagenome: 34 (9) SCZ-16S rRNA: 35 (11) Control-16S rRNA: 35 (11) | SCZ-Metagenome: 20/20 Control-Metagenome: 20/20 SCZ-16S rRNA: 28/16 Control-16S rRNA: 21/16 | SCZ-Metagenome: - Control-Metagenome: - SCZ-16S rRNA: 22(3.21) Control-16S rRNA: 23.09 (3.71) | NA | DSM-V | - | Feces | 16S rRNA amplicon sequencing Metagenomic sequencing | 16S rRNA: V4 | SCZ and Control |
| Ma X 2020[20] | China | SCZ (n=125) Control (n=69) | SCZ: 24.1 (6.1)  Control: 23.1 (3.2) | SCZ: 68/57 Control: 37/32 | - | Antipsychotics | DSM-IV | - | Feces | 16S rRNA amplicon sequencing | V4 | First-episode drug-naïve SCZ and Control Antipsychotic-treated SCZ and Control |
| Li S 2020[21] | China | SCZ (n=82) Control (n=80) | SCZ: 42.1 (13.1)  Control: 41.0 (14.3) | SCZ: 46/36 Control: 39/41 | SCZ: 24.48 (4.33) Control: 23.03 (3.05) | NA | DSM-IV-TR | SCZ: 59.1 (18.1) | Feces | 16S rRNA amplicon sequencing | V4 | SCZ and Control |
| Zheng P 2019[22] | China | SCZ (n=63) Control (n=69) | SCZ: 43.4 (1.6) Control: 39.9 (1.6) | SCZ: 42/21 Control: 36/33 | SCZ: 22.90 (0.32) Control: 23.16 (0.33) | NA | DSM-IV | SCZ: 71.8 (1.8) | Feces | 16S rRNA amplicon sequencing  Metagenomic sequencing | 16S rRNA: V3-V4 | SCZ and Control |
| Nguyen TT 2019[23] | USA | SCZ (n=25) Control (n=25) | SCZ: 52.9 (11.2) Control: 54.7 (10.7) | SCZ: 14/11 Control: 15/10 | SCZ: 31.8 (5.4) Control: 28.9 (4.0) | NA | DSM-IV-TR | - | Feces | 16S rRNA amplicon sequencing | V4 | SCZ and Control |
| Shen Y 2018[24] | China | SCZ (n=64) Control (n=53) | SCZ: 42 (11) Control: 39 (14) | SCZ: 36/28 Control: 35/18 | SCZ: 23.49 (3.8) Control: 23.14 (2.8) | NA | ICD-10 | SCZ ＜ 60 | Feces | 16S rRNA amplicon sequencing | V3-V4 | SCZ and Control |
| Schwarz E 2017[25] | Germany | SCZ (n=28) Control (n=16) | SCZ: 25.9 (5.5) Control: 27.8 (6.0) | SCZ: 16/12 Control: 8/8 | SCZ: 23.8 (4.3) Control: 23.9 (3.1) | NA | DSM-IV | - | Feces | Metagenomic sequencing | - | SCZ and Control |
| He Y 2018[26] | China | Ultra-high risk SCZ (n=19) High risk SCZ (n=81) Control (n=69) | Ultra-high risk SCZ: 20.4 (4.5) High risk SCZ: 21.6 (5.7) Control: 23.1 (3.8) | Ultra-high risk SCZ: 15/4 High risk SCZ: 41/40 Control: 37/32 | - | NA | DSM-IV-TR | - | Feces | 16S rRNA amplicon sequencing | V4 | Ultra-high risk SCZ and Control High risk SCZ and Control |
| Wang Z 2023[27] | China | First-episode drug-naïve SCZ (n=127) Control (n=92) | First-episode drug-naïve SCZ: 21.6 (7.5) Control: 22.9 (2.5) | First-episode drug-naïve SCZ: 60/67 Control: 35/57 | First-episode drug-naïve SCZ: 21.49 (4.05) Control: 21.30 (3.09) | NA | DSM-IV | First-episode drug-naïve SCZ: 84.6 (13.8) | Feces | Metagenomic sequencing | - | First-episode drug-naïve SCZ and Control |
| Thirion F 2023[28] | Denmark | SCZ (n=132) Control (n=132) | SCZ: 41 (12) Control: 41 (12) | SCZ: 59/73 Control: 57/75 | SCZ: 35 (6.2) Control: 24 (3.8) | NA | - | - | Feces | Metagenomic sequencing | - | SCZ and Control |
| Li H 2023[29] | China | SCZ (n=68) Control (n=72) | SCZ: 43.3 (12.8) Control: 42.2 (14.5) | SCZ: 43/25 Control: 33/39 | SCZ: 23.9 (4.1) Control: 23.0 (3.1) | NA | DSM-IV | SCZ: 59.4 (18.6) | Feces | 16S rRNA amplicon sequencing | V4 | SCZ and Control |
| Gokulakrishnan K 2023[30] | India | SCZ + risperidone (n=20) Drug-naïve SCZ (n=20) Control (n=20) | SCZ + risperidone: 34 (9.3) Drug-naïve SCZ: 33 (8.1) Control: 32 (5.6) | SCZ + risperidone: 8/12 Drug-naïve SCZ: 9/11 Control: 11/9 | SCZ + risperidone: 25.40 (5.3) Drug-naïve SCZ: 22.31 (5.0) Control: 24.71 (5.5) | Risperidone | DSM-V | - | Feces | 16S rRNA amplicon sequencing | V3-V4 | SCZ + risperidone and Control Drug-naïve SCZ and Control |

**References**

1. Yan, F., et al., *A comparative study to determine the association of gut microbiome with schizophrenia in Zhejiang, China.* BMC Psychiatry, 2022. 22(1): p. 731.

2. Wang, X.P., et al., *[Variations in fecal microbiota of first episode schizophrenia associated with clinical assessment and serum metabolomics].* Beijing Da Xue Xue Bao Yi Xue Ban, 2022. 54(5): p. 863-873.

3. O'Donnell, M., et al., *The Role of the Microbiome in the Metabolic Health of People with Schizophrenia and Related Psychoses: Cross-Sectional and Pre-Post Lifestyle Intervention Analyses.* Pathogens, 2022. 11(11).

4. Misiak, B., et al., *Gut microbiota alterations in stable outpatients with schizophrenia: findings from a case-control study.* Acta Neuropsychiatr, 2023. 35(3): p. 147-155.

5. Ma, Q., et al., *Characterizing serum amino acids in schizophrenic patients: Correlations with gut microbes.* J Psychiatr Res, 2022. 153: p. 125-133.

6. Ling, Z., et al., *Fecal Dysbiosis and Immune Dysfunction in Chinese Elderly Patients With Schizophrenia: An Observational Study.* Front Cell Infect Microbiol, 2022. 12: p. 886872.

7. Liang, Y., et al., *Schizophrenia Patients With Prevotella-Enterotype Have a Higher Risk of Obesity.* Front Psychiatry, 2022. 13: p. 864951.

8. Gao, Y., et al., *Integrated untargeted fecal metabolomics and gut microbiota strategy for screening potential biomarkers associated with schizophrenia.* J Psychiatr Res, 2022. 156: p. 628-638.

9. Fan, Y., et al., *Multi-Omics Analysis Reveals Aberrant Gut-Metabolome-Immune Network in Schizophrenia.* Front Immunol, 2022. 13: p. 812293.

10. Chen, Y.H., et al., *Gut microbial signatures and differences in bipolar disorder and schizophrenia of emerging adulthood.* CNS Neurosci Ther, 2023. 29 Suppl 1(Suppl 1): p. 5-17.

11. Zhu, C., et al., *Association Between Abundance of Haemophilus in the Gut Microbiota and Negative Symptoms of Schizophrenia.* Front Psychiatry, 2021. 12: p. 685910.

12. Yuan, X., et al., *Gut microbial biomarkers for the treatment response in first-episode, drug-naïve schizophrenia: a 24-week follow-up study.* Transl Psychiatry, 2021. 11(1): p. 422.

13. Miao, Y., et al., *Effect of the correlation between gut microbiota and folic acid in first-episode schizophrenia.* Zhonghua Yi Xue Za Zhi, 2021. 101(37): p. 3012-3017.

14. Manchia, M., et al., *Involvement of Gut Microbiota in Schizophrenia and Treatment Resistance to Antipsychotics.* Biomedicines, 2021. 9(8).

15. Nguyen, T.T., et al., *Gut microbiome in Schizophrenia: Altered functional pathways related to immune modulation and atherosclerotic risk.* Brain Behav Immun, 2021. 91: p. 245-256.

16. Li, S., et al., *The gut microbiome is associated with brain structure and function in schizophrenia.* Sci Rep, 2021. 11(1): p. 9743.

17. Zhu, F., et al., *Metagenome-wide association of gut microbiome features for schizophrenia.* Nat Commun, 2020. 11(1): p. 1612.

18. Zhang, X., et al., *Analysis of gut mycobiota in first-episode, drug-naïve Chinese patients with schizophrenia: A pilot study.* Behav Brain Res, 2020. 379: p. 112374.

19. Xu, R., et al., *Altered gut microbiota and mucosal immunity in patients with schizophrenia.* Brain Behav Immun, 2020. 85: p. 120-127.

20. Ma, X., et al., *Alteration of the gut microbiome in first-episode drug-naïve and chronic medicated schizophrenia correlate with regional brain volumes.* J Psychiatr Res, 2020. 123: p. 136-144.

21. Li, S., et al., *Altered gut microbiota associated with symptom severity in schizophrenia.* PeerJ, 2020. 8: p. e9574.

22. Zheng, P., et al., *The gut microbiome from patients with schizophrenia modulates the glutamate-glutamine-GABA cycle and schizophrenia-relevant behaviors in mice.* Sci Adv, 2019. 5(2): p. eaau8317.

23. Nguyen, T.T., et al., *Differences in gut microbiome composition between persons with chronic schizophrenia and healthy comparison subjects.* Schizophr Res, 2019. 204: p. 23-29.

24. Shen, Y., et al., *Analysis of gut microbiota diversity and auxiliary diagnosis as a biomarker in patients with schizophrenia: A cross-sectional study.* Schizophr Res, 2018. 197: p. 470-477.

25. Schwarz, E., et al., *Analysis of microbiota in first episode psychosis identifies preliminary associations with symptom severity and treatment response.* Schizophr Res, 2018. 192: p. 398-403.

26. He, Y., et al., *Gut microbiome and magnetic resonance spectroscopy study of subjects at ultra-high risk for psychosis may support the membrane hypothesis.* Eur Psychiatry, 2018. 53: p. 37-45.

27. Wang, Z., et al., *Multiomics Analyses Reveal Microbiome-Gut-Brain Crosstalk Centered on Aberrant Gamma-Aminobutyric Acid and Tryptophan Metabolism in Drug-Naïve Patients with First-Episode Schizophrenia.* Schizophr Bull, 2023.

28. Thirion, F., et al., *Alteration of Gut Microbiome in Patients With Schizophrenia Indicates Links Between Bacterial Tyrosine Biosynthesis and Cognitive Dysfunction.* Biol Psychiatry Glob Open Sci, 2023. 3(2): p. 283-291.

29. Li, H., et al., *Association of serum homocysteine levels with intestinal flora and cognitive function in schizophrenia.* J Psychiatr Res, 2023. 159: p. 258-265.

30. Gokulakrishnan, K., et al., *Comparison of gut microbiome profile in patients with schizophrenia and healthy controls - A plausible non-invasive biomarker?* J Psychiatr Res, 2023. 162: p. 140-149.

**Table S6.** Microbiota diversity in patients with schizophrenia.

| **Study** | **Comparison groups** | **Alpha diversity assessment** | **Alpha diversity alteration** | **Beta diversity assessment** | **Beta diversity alteration** |
| --- | --- | --- | --- | --- | --- |
| Yan F 2022 | SCZ and Control | Shannon | Decreased | Bray-Curtis | Significantly different |
|  |  | Simpson | Decreased |  |  |
|  |  | Observed features | Decreased |  |  |
| Wang X 2022 | First-episode SCZ (baseline) and Control (baseline) | Observed features | NS | Unweighted UniFrac | Significantly different |
|  |  | Shannon | NS |  |  |
|  |  | Simpson | NS |  |  |
|  |  | Chao1 | NS |  |  |
|  |  | ACE | NS |  |  |
|  |  | Goods coverage | Increased |  |  |
|  |  | PD whole tree | NS |  |  |
|  | First-episode SCZ + 4-week risperidone and Control (baseline) | Observed features | NS | Unweighted UniFrac | Significantly different |
|  |  | Shannon | NS |  |  |
|  |  | Simpson | NS |  |  |
|  |  | Chao1 | Increased |  |  |
|  |  | ACE | Increased |  |  |
|  |  | Goods coverage | Increased |  |  |
|  |  | PD whole tree | NS |  |  |
| O'Donnell M 2022 | SCZ + 1-year clozapine and Control | Gini | NS | Bray-Curtis | Significantly different |
|  |  | Evenness | NS |  |  |
|  |  | Chao1 | NS |  |  |
|  |  | Chao2 | NS |  |  |
|  |  | Inverse Simpson | NS |  |  |
|  |  | Simpson | NS |  |  |
|  |  | Shannon | NS |  |  |
|  |  | Observed features | NS |  |  |
|  | First-episode SCZ and Control | Gini | NS | Bray-Curtis | Significantly different |
|  |  | Evenness | NS |  |  |
|  |  | Chao1 | NS |  |  |
|  |  | Chao2 | NS |  |  |
|  |  | Inverse Simpson | NS |  |  |
|  |  | Simpson | NS |  |  |
|  |  | Shannon | NS |  |  |
|  |  | Observed features | NS |  |  |
| Misiak B 2022 | SCZ and Control | PD whole tree | NS | Bray-Curtis | Not reported |
|  |  | Shannon | NS |  |  |
|  |  | Pielou | NS |  |  |
| Ma Q 2022 | SCZ with low-level serum amino acid and Control | Shannon | Increased | Bray-Curtis | Significantly different |
|  |  | Simpson | Increased |  |  |
|  | SCZ with mid-level serum amino acid group and Control | Shannon | NS | Bray-Curtis | Not reported |
|  |  | Simpson | NS |  |  |
| Ling Z 2022 | Elderly SCZ and Control | ACE | NS | Unweighted UniFrac | Significantly different |
|  |  | Chao1 | NS |  |  |
|  |  | Shannon | NS |  |  |
|  |  | Simpson | NS |  |  |
|  |  | Evenness | NS |  |  |
|  |  | PD whole tree | NS |  |  |
| Liang Y 2022 | SCZ and Control | Not reported | Not reported | Not reported | Not reported |
| Gao Y 2022 | Paranoid SCZ and Control | Observed features | Not reported | Weighted UniFrac | Significantly different |
|  |  | Shannon | Decreased |  |  |
|  |  | Simpson | Decreased |  |  |
|  |  | Chao1 | Not reported |  |  |
|  | Undifferentiated SCZ and Control | Observed features | Not reported | Weighted UniFrac | Significantly different |
|  |  | Shannon | Not reported |  |  |
|  |  | Simpson | Decreased |  |  |
|  |  | Chao1 | Not reported |  |  |
| Fan Y 2022 | SCZ and Control | Shannon | Increased | PLS-DA | Significantly different |
| Chen Y 2022 | SCZ and Control | Observed features | NS | Bray-Curtis | NS |
|  |  | Chao1 | NS |  |  |
|  |  | ACE | NS |  |  |
|  |  | Simpson | NS |  |  |
|  |  | Shannon | NS |  |  |
|  |  | PD whole tree | NS |  |  |
| Zhu C 2021 | Acute SCZ and Control | Chao1 | NS | Unweighted UniFrac | NS |
|  |  | Ace | NS |  |  |
|  |  | Shannon | NS |  |  |
|  |  | Simpson | NS |  |  |
|  |  | Observed features | NS |  |  |
|  |  | Goods coverage | NS |  |  |
|  | Remission SCZ and Control | Chao1 | NS | Unweighted UniFrac | NS |
|  |  | Ace | NS |  |  |
|  |  | Shannon | NS |  |  |
|  |  | Simpson | NS |  |  |
|  |  | Observed features | NS |  |  |
|  |  | Goods coverage | NS |  |  |
| Yuan X 2021 | SCZ and Control | Shannon | Increased | Bray-Curtis | Significantly different |
|  |  | Simpson | Increased |  |  |
|  |  | PD whole tree | Increased |  |  |
|  |  | Observed features | NS | Unweighted UniFrac | Significantly different |
|  |  | Chao1 | NS |  |  |
|  |  | ACE | NS |  |  |
| Miao Y 2021 | First-episode SCZ and Control | Not reported | Not reported | Not reported | Not reported |
| Manchia M 2021 | SCZ and Control | Chao1 | Not reported | Not reported | Not reported |
|  |  | Shannon | NS |  |  |
|  | SCZ with treatment responsive and Control | Not reported | Not reported | Not reported | Not reported |
|  | SCZ with treatment resistant and Control | Not reported | Not reported | Not reported | Not reported |
| Nguyen TT 2021 | SCZ and Control | Observed features | NS | Bray-Curtis, Unweighted UniFrac | Significantly different Significantly different |
|  |  | Shannon | NS |  |  |
|  |  | PD whole tree | NS |  |  |
| Li S 2021 | SCZ and Control | PD whole tree | NS | Bray-Curtis | Significantly different |
|  |  | Observed features | NS |  |  |
|  |  | Shannon | NS |  |  |
|  |  | Evenness | NS |  |  |
| Zhu F 2020 | SCZ and Control | Shannon | Increased | Bray-Curtis | Significantly different |
| Zhang X 2019 | SCZ and Control | Chao1 | NS | Bray-Curtis, Weighted UniFrac,  Unweighted UniFrac | NS NS NS |
|  |  | Shannon | NS |  |  |
|  |  | Simpson | NS |  |  |
|  |  | Sobs | NS |  |  |
| Xu R 2020 | SCZ and Control | Chao1 | Decreased | Nonmetric multidimensional scale analysis | NS |
| Ma X 2020 | First-episode drug-naïve SCZ and Control | Shannon | NS | Weighted UniFrac,  Unweighted UniFrac | NS NS |
|  |  | Chao1 | NS |  |  |
|  | Antipsychotic-treated SCZ and Control | Shannon | Decreased | Weighted UniFrac,  Unweighted UniFrac | NS NS |
|  |  | Chao1 | Decreased |  |  |
| Li S 2020 | SCZ and Control | Evenness | NS | Bray-Curtis | Significantly different |
|  |  | PD whole tree | NS |  |  |
|  |  | Observed features | NS |  |  |
|  |  | Shannon | NS |  |  |
| Zheng P 2019 | SCZ and Control | ACE | Decreased | PLS-DA | NS |
|  |  | Chao1 | Decreased |  |  |
|  |  | Shannon | Decreased |  |  |
| Nguyen TT 2019 | SCZ and Control | Observed features | NS | Unweighted UniFrac, Bray-Curtis | NS NS |
|  |  | Shannon | NS |  |  |
|  |  | PD whole tree | NS |  |  |
| Shen Y 2018 | SCZ and Control | Observed features | NS | Unweighted UniFrac | Significantly different |
|  |  | Shannon | NS |  |  |
|  |  | Simpson | NS |  |  |
|  |  | ACE | NS |  |  |
|  |  | Chao1 | NS |  |  |
|  |  | PD whole tree | NS |  |  |
| Schwarz E 2017 | SCZ and Control | Not reported | Not reported | Not reported | Not reported |
| He Y 2018 | Ultra-high risk SCZ and Control | Observed features | NS | Canberra distance matrix | Significantly different |
|  |  | Shannon | NS |  |  |
|  | High risk SCZ and Control | Observed features | NS | Canberra distance matrix | Significantly different |
|  |  | Shannon | NS |  |  |
| Wang Z 2023 | First-episode drug-naïve SCZ and Control | Shannon | Decreased | Bray-Curtis, Hellinger distance | Not reported Not reported |
|  |  | Richness | Decreased |  |  |
| Thirion F 2023 | SCZ and Control | Shannon | Decreased | Bray-Curtis | Significantly different |
| Li H 2023 | SCZ and Control | Shannon | NS | Bray-Curtis | Significantly different |
|  |  | Observed features | NS |  |  |
|  |  | Evenness | NS |  |  |
|  |  | PD whole tree | NS |  |  |
| Gokulakrishnan K 2023 | SCZ + risperidone and Control | Shannon | NS | Bray-Curtis | Significantly different |
|  |  | Simpson | NS |  |  |
|  |  | Chao1 | NS |  |  |
|  | Drug-naïve SCZ and Control | Shannon | NS | Bray-Curtis | Significantly different |
|  |  | Simpson | NS |  |  |
|  |  | Chao1 | NS |  |  |

Abbreviations: NS, no significant difference.

**Table S7.** The number of the differential gut microbiota and candidate taxa in patients with schizophrenia and subgroups.

| **Group** | **No. of studies** | **No. of differential taxa (no. of candidate taxa) after removing duplicates** | | | | | | |
| --- | --- | --- | --- | --- | --- | --- | --- | --- |
|  |  | **Phylum** | **Class** | **Order** | **Family** | **Genus** | **Species** | **Total** |
| Entire patients group | 30 | 15 (5) | 16 (4) | 29 (6) | 62 (18) | 174 (53) | 132 (2) | 428 (88) |
| Mildly ill group | 2 | 0 | 0 | 0 | 0 | 5 (0) | 21 (0) | 26 (0) |
| Moderately ill group | 8 | 7 (2) | 8 (1) | 14 (2) | 25 (3) | 96 (12) | 42 (0) | 192 (20) |
| 16S rRNA amplicon sequencing group | 23 | 14 (5) | 16 (3) | 22 (4) | 56 (14) | 152 (49) | 63 (0) | 323 (75) |
| Metagenomic sequencing group | 6 | 4 (1) | 5 (0) | 11 (0) | 16 (0) | 48 (0) | 77 (0) | 161 (1) |
| Drug treated group | 4 | 3 (0) | 0 | 0 | 7 (0) | 29 (0) | 20 (0) | 59 (0) |
| Untreated group | 30 | 14 (5) | 16 (4) | 29 (6) | 59 (18) | 169 (45) | 118 (2) | 405 (80) |
| Chinese group | 22 | 13 (4) | 14 (3) | 27 (5) | 54 (17) | 145 (48) | 79 (2) | 332 (79) |
| Youth group | 10 | 6 (3) | 8 (2) | 12 (2) | 23 (5) | 85 (12) | 54 (0) | 188 (24) |
| Elderly group | 12 | 11 (4) | 11 (1) | 20 (3) | 46 (8) | 105 (24) | 32 (0) | 225 (40) |


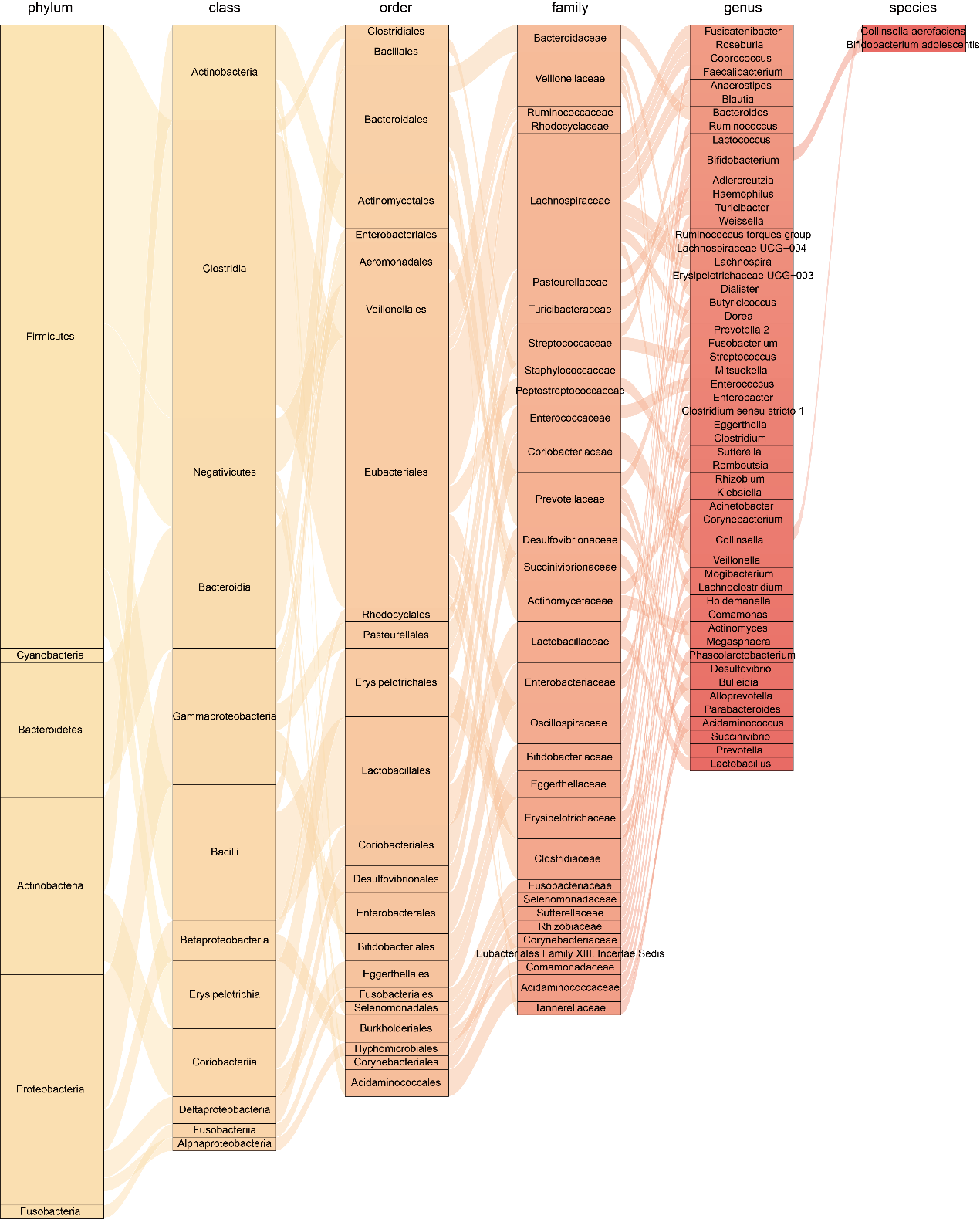


**Figure S1**


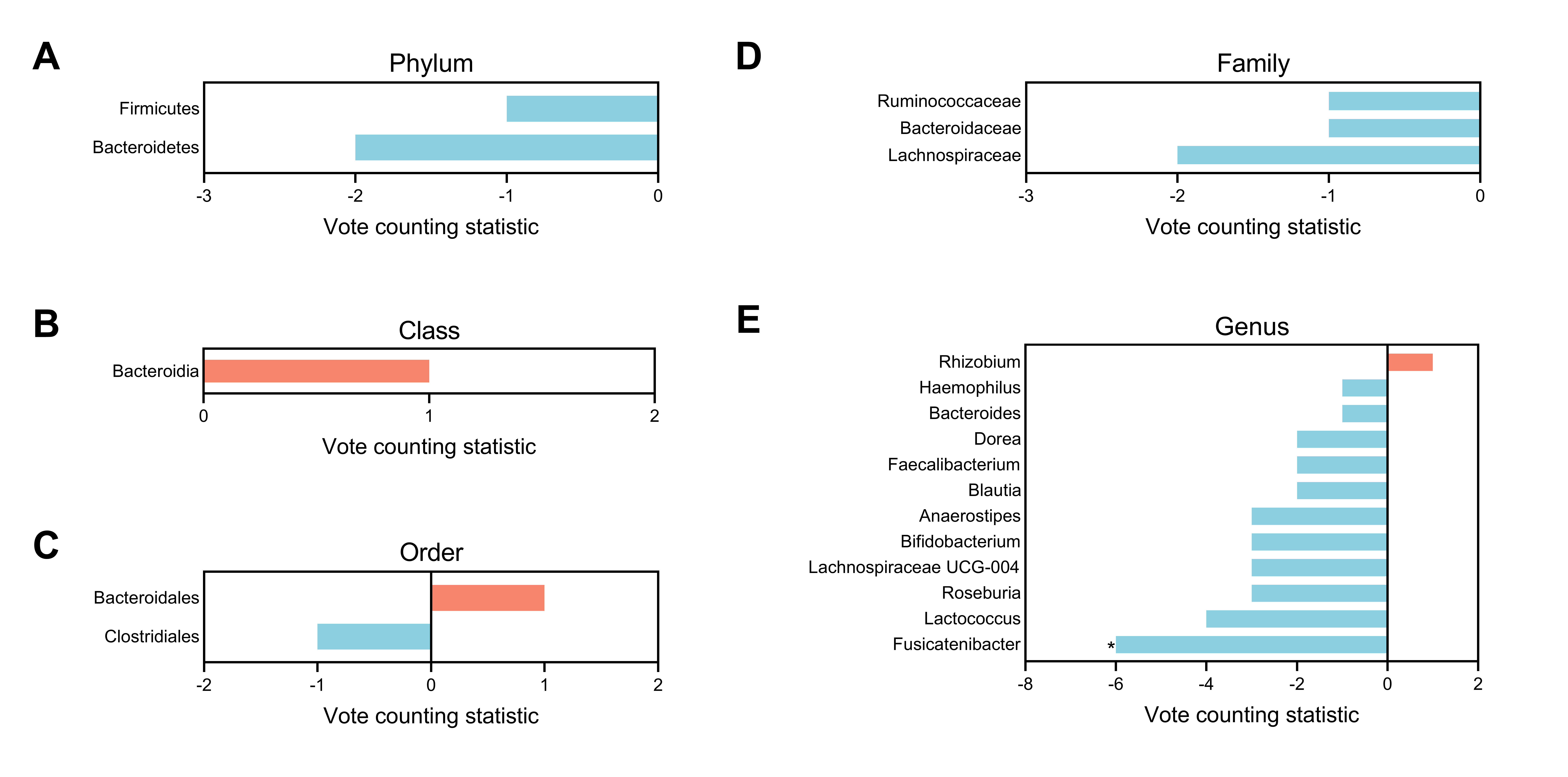


**Figure S2**

**
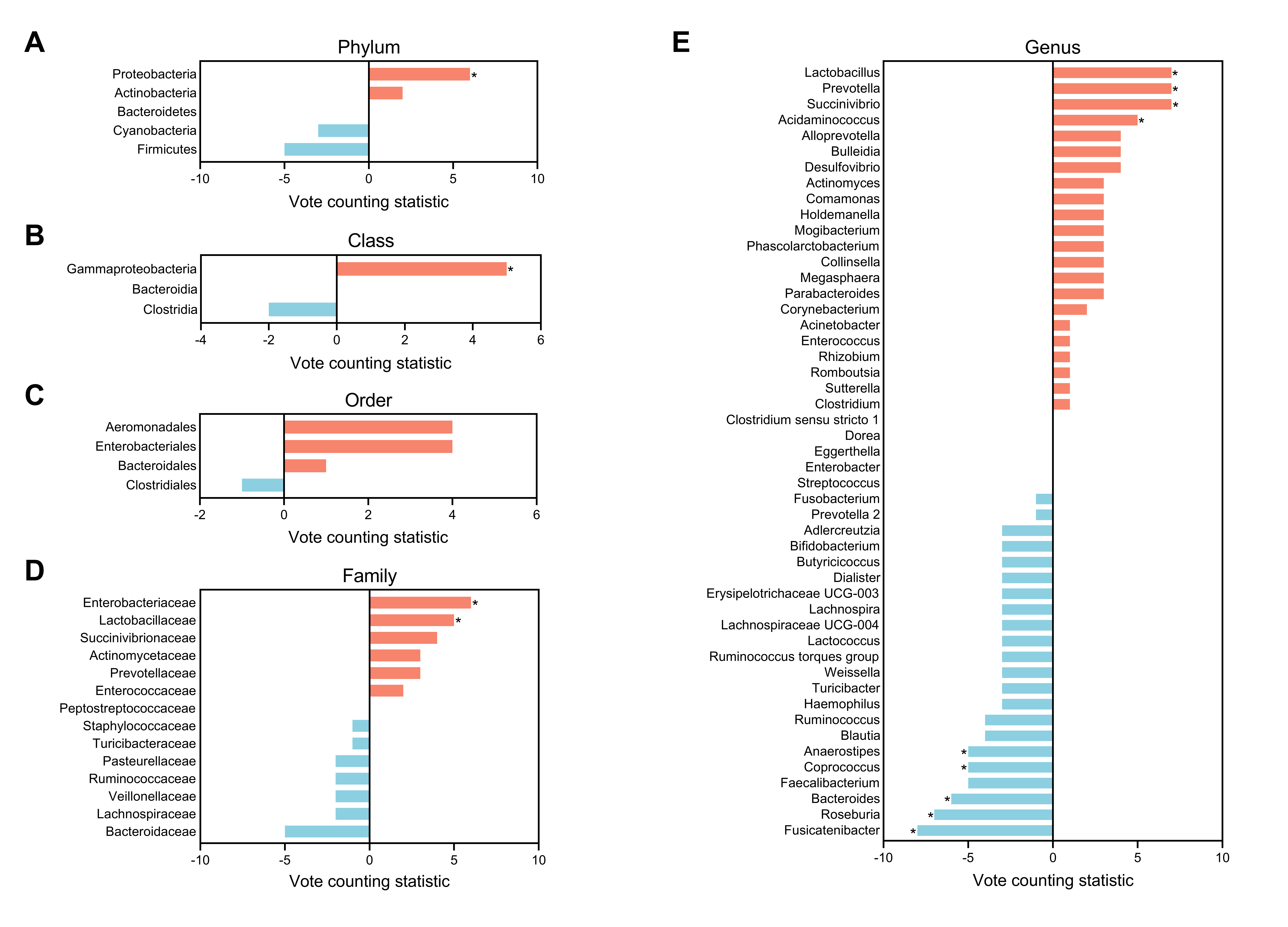
**

**Figure S3**





**Figure S4**





**Figure S5**

**
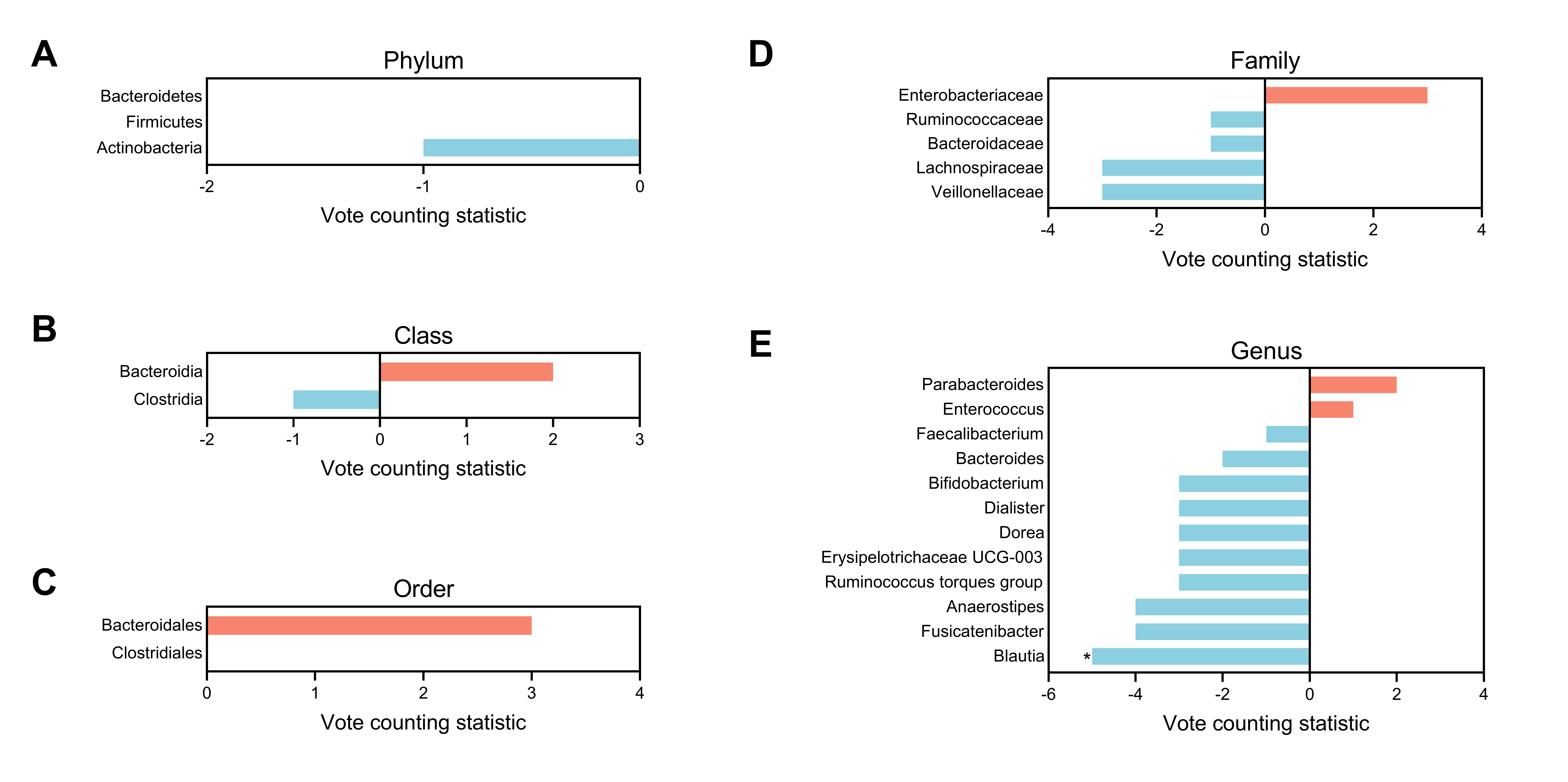
**

**Figure S6**

**
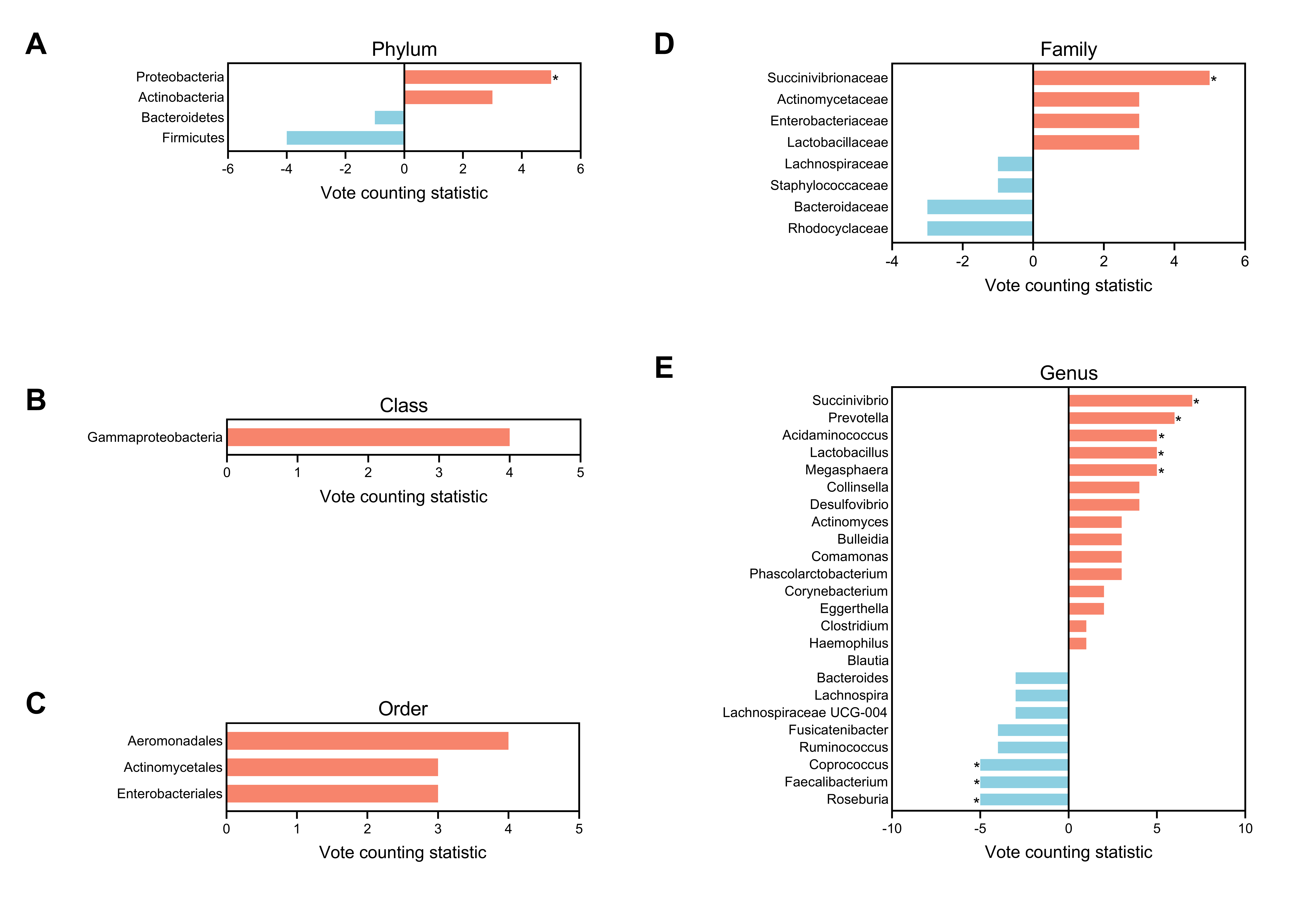
**

**Figure S7**
